# Evidence of reduced recombination rate in human regulatory domains

**DOI:** 10.1186/s13059-017-1308-x

**Published:** 2017-10-20

**Authors:** Yaping Liu, Abhishek Sarkar, Pouya Kheradpour, Jason Ernst, Manolis Kellis

**Affiliations:** 1 Computer Science and Artificial Intelligence Lab (CSAIL), Massachusetts Institute of Technology, Massachusetts, USA; 2 Broad Institute of MIT and Harvard, Cambridge, Massachusetts USA; 3 Department of Biological Chemistry, David Geffen School of Medicine, University of California at Los Angeles, California, USA

**Keywords:** Recombination rate, Regulatory domain, DNA methylation

## Abstract

**Background:**

Recombination rate is non-uniformly distributed across the human genome. The variation of recombination rate at both fine and large scales cannot be fully explained by DNA sequences alone. Epigenetic factors, particularly DNA methylation, have recently been proposed to influence the variation in recombination rate.

**Results:**

We study the relationship between recombination rate and gene regulatory domains, defined by a gene and its linked control elements. We define these links using expression quantitative trait loci (eQTLs), methylation quantitative trait loci (meQTLs), chromatin conformation from publicly available datasets (Hi-C and ChIA-PET), and correlated activity links that we infer across cell types. Each link type shows a “recombination rate valley” of significantly reduced recombination rate compared to matched control regions. This recombination rate valley is most pronounced for gene regulatory domains of early embryonic development genes, housekeeping genes, and constitutive regulatory elements, which are known to show increased evolutionary constraint across species. Recombination rate valleys show increased DNA methylation, reduced doublestranded break initiation, and increased repair efficiency, specifically in the lineage leading to the germ line. Moreover, by using only the overlap of functional links and DNA methylation in germ cells, we are able to predict the recombination rate with high accuracy.

**Conclusions:**

Our results suggest the existence of a recombination rate valley at regulatory domains and provide a potential molecular mechanism to interpret the interplay between genetic and epigenetic variations.

**Electronic supplementary material:**

The online version of this article (doi:10.1186/s13059-017-1308-x) contains supplementary material, which is available to authorized users.

## Background

Variation in recombination rates in humans and other diploid organisms can be shaped by evolutionary and molecular processes [1], but these forces are only partially understood. High-resolution human recombination maps have been estimated using both parent–offspring transmission [2, 3] and patterns of linkage disequilibrium (LD) [4–7]. These have revealed localized regions with higher or lower recombination rates, known as recombination hotspots and coldspots, respectively [5]. Sequences analysis has shown that human recombination hotspots are associated with a number of sequence features such as PRDM9 binding motifs [8], CpG islands, and GC-rich repeats [4, 5, 9], and that recombination coldspots are associated with repetitive elements, transcribed regions, and telomeres [5, 6].

Outside recombination hotspots, differences in epigenomic signatures are associated with differences in recombination rate [10, 11]. In particular, the level of DNA methylation, primarily established at prophase I when recombination occurs [12], is reported to be positively correlated with recombination rate [11]. A causal effect of DNA methylation on recombination rate was established using a methylation-deficient strain of *Arabidopsis*, which showed reduction of recombination rate in euchromatic regions [13, 14].

## Results

### Gene regulatory domains defined using expression and methylation quantitative trait loci show a recombination rate valley

We examined the relationship between human recombination rate and regulatory domains, defined as the genomic region spanned by a gene and the regulatory regions linked to its promoter element within the same chromosome. Recombination rates were estimated using the 1000 Genomes genetic map [4], then related to gene regulatory domains using four types of links.

We first used genetic links based on expression quantitative trait loci (eQTLs) and methylation quantitative trait loci (meQTLs). These consist of 248,856 eQTL links between regulatory regions and transcription start sites (TSSs) of target genes, defined in whole blood using 168 individuals profiled by the Gene-Tissue Expression (GTEx) Consortium [15], and 809,577 meQTL links between regulatory regions and CpG methylation measured in human brain, primarily in promoter regions, in the ROS/MAP cohort of 575 individuals [16].

**Fig. 1:**
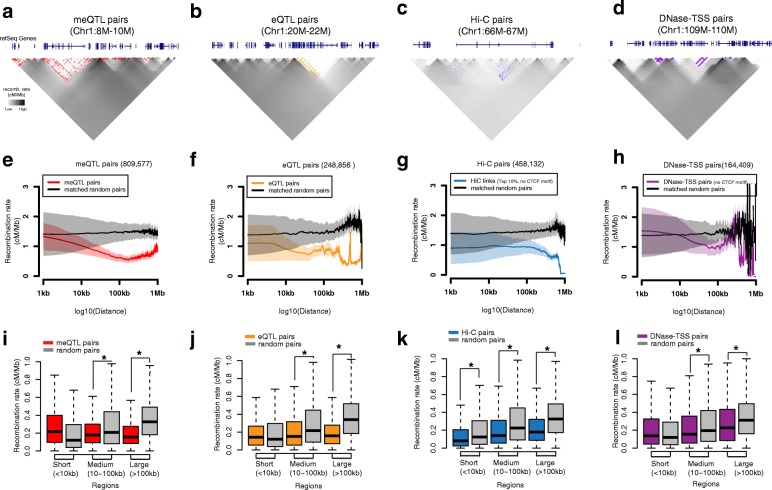
Recombination valleys within genetic, physical, and activity links. Example genomic regions to show the enrichment of **a** meQTL pairs (*red*), **b** eQTL pairs (*orange*), **c** top 10% Hi-C pairs (observed/expected (O/E), no CTCF motif, *blue*), **d** DNase–TSS pairs (no CTCF motif, *purple*) in low recombination rate regions. Each pixel represents a 10-kb segment (for Hi-C, a 1-kb segment) and darker color indicates higher recombination rate between two segments. *Colored dots* at each pixel indicate the genetic or physical links that exist between two genomic segments. The average recombination rate within **e** meQTL pairs (*red*), **f** eQTL pairs (*orange*), **g** top 10% of Hi-C pairs (O/E, no CTCF motif, *blue*), **h** DNase–TSS pairs (no CTCF motif, *purple*) are significantly lower than the matched random intervals. *Colored lines* represent the mean recombination rate at each interval distance between genomic features, while *black links* represent the mean value in matched random intervals. Shaded regions represent the 95% confidence interval (mean ± standard deviation × 1.96/10). Recombination rate in three different genomic scales in **i** meQTL pairs, **j** eQTL pairs, **k** top 10% of Hi-C pairs (O/E, no CTCF motif), **l** DNase–TSS pairs (no CTCF motif). Comparisons with *p* < 1e^−4^ (two-way paired Mann–Whitney U test) are marked with an *asterisk*

We found that the intervals between eQTLs and their target genes, and between meQTLs and their target methylation probes, showed substantial decreases in recombination rate (Fig. 1a, b). We evaluated intervals at three distance ranges, consisting of short (1–10 kb), intermediate (10–100 kb), and long (100 kb–1 Mb) distances. The effect was most pronounced for links of intermediate and long distances, which showed consistently lower recombination rates compared to random intervals, a phenomenon we call a “recombination rate valley” (Fig. 1e, f; Additional file 1: Figure S1a, b). In short intervals, the accuracy of recombination rate estimation is affected by variable SNP density in different genomic regions and genetic maps. Therefore, we did not observe consistent recombination rate valleys within short-range intervals. To evaluate the statistical significance of the observed recombination rate valleys, we sampled the same number of random genomic regions with the same physical length in the same chromosome (details in “Methods”). As an additional comparator, we sampled the same number of random SNP-TSS/SNP-CpG pairs (details in “Methods”). We found significant decreases in recombination rate at both intermediate and long distances for both eQTL and meQTL links (Fig. 1i, j) in both cases (two-way paired Mann–Whitney U test with random matched intervals and permutation test with random combinations of SNP-TSS/SNP-CpG, *p* < 1e^−4^).

We next confirmed that our observation in genetics-based links is not an artifact of linkage disequilibrium (LD), which is a possibility as more genetic links are found at regions with stronger LD. First, we only kept the eQTL/meQTL with the most significant *p* value for each gene/CpG. We further pruned these “best” eQTL/meQTL links by excluding multiple counts from the same genomic region (details in Additional file 2: Supplemental method 1) and consistently found significant recombination rate valleys in the regions defined by genetic links (Additional file 1: Figure S2a–c, f–h, k–m). Second, we made a small random shift around the best genetic links and found a slightly but significantly higher recombination rate (Additional file 1: Figure S3; Additional file 2: Supplemental method 2). These observations suggest that causal genetics links show lower recombination rates within LD.

We further evaluated whether our observation held in independent datasets and with varying analytical parameters. First, we repeated the analysis using 16 additional tissues and cell lines from the GTEx Consortium [15], the Multiple Tissue Human Expression Resource (MuTHER) Consortium [17], and Genetic European Variation in Health and Disease (gEUVADIS) Consortium [18] and again consistently found a significant recombination rate valley in the regions defined by genetic links (Additional file 1: Figure S4). Second, we repeated the analysis varying thresholds for the false discovery rate (FDR) of eQTLs and meQTLs, and consistently found recombination rate valleys. The strongest reduction in recombination rate was found at the most stringent FDR thresholds for eQTL and meQTL discovery, indicating that, with higher link confidence thresholds, the signal becomes stronger (Additional file 1: Figure S5a, b, e, f, i, j). Third, we repeated the analysis on genetic maps estimated by the HapMap project [19] and by deCODE Genetics [2, 3] and found the results largely unchanged (Additional file 1: Figure S6a, b, e, f, i, l). To account for sequence biases, we used a rejection sampling approach to generate matched random intervals, with equal GC content, CpG density, SNP density, and PRDM9 motif density. We found the results robust to this more stringent matching (Additional file 1: Figure S7a, b, e, f, i, j, m, n, q, r). Since it is computationally very expensive to generate random matched controls by a rejection sampling approach in high dimensional space, we implemented a k-d tree data structure to organize all possible 1-kb to 1-Mb random intervals in the genome and searched with even more stringent matching criteria, including additional features of gene density and distance to TSS (details in “Methods”; Additional file 1: Figure S7u, v, y, z, ac–af). To account for the decreased recombination rate in transcribed regions, we excluded intervals within 2 kb of gene annotations in GENCODE v19 and still found recombination rate valleys in intergenic meQTL/eQTL links compared with random intervals that were generated with our stringent matched criteria. The conclusion did not change when we averaged the recombination rate from only non-coding bases (Additional file 1: Figure S8a, b, e, f, i, j, m–p).

### Gene regulatory domains by chromosome conformation show a recombination valley

In addition to genetic links, we used 458,132 links between genomic regions in close proximity when folded in the three-dimensional nucleus, based on high-throughput chromosome conformation capture (Hi-C) measured in the GM12878 cell line [20]. We found that the recombination rate within regulatory domains defined by Hi-C was also significantly lower (two-way paired Mann–Whitney U test and permutation test, *p* < 1e^−4^) at both intermediate and long distances compared with two different sets of random intervals (Fig. 1c, g, k; Additional file 1: Figure S1c). This property held specifically for Hi-C links not interrupted by CTCF motifs [21], consistent with the role of CTCF loops as defining regulatory domain boundaries [20] (Fig. 1g, k; Additional file 1: Figure S11o, p). We also excluded CTCF motifs from random matched intervals and still found significant depletions (Additional file 1: Figure S12a, b).

To avoid the bias introduced by relatively more Hi-C links from the domains with lower recombination rate, we generated the matched random intervals only within the same loops detected by Hi-C computational unbiased peak search (HiCCUPS loops) and still found recombination rate valleys (Additional file 1: Figure S9). We also pruned the Hi-C links by excluding multiple counts of each genomic region and consistently found recombination rate valleys (Additional file 1: Figure S2d, i, n; Additional file 2: Supplementary method 1). We next varied the threshold for Hi-C links (no CTCF motif) included in the analysis and continued to observe recombination rate valleys (Additional file 1: Figure S5c, g). We also repeated the analysis in different genetic maps (Additional file 1: Figure S6c, g) and compared this with more stringent matched random intervals in the whole genome by two methods (Additional file 1: Figure S7c, g, k, o, s, w, aa) and in non-coding and intergenic regions (Additional file 1: Figure S8c, g, k). We combined Hi-C and eQTL evidence available in the same cell type (lymphoblastoid cell lines (LCLs), including GM12878) [17, 20] and found that the depletion in the recombination rate became even more pronounced (Additional file 1: Figure S10; two-way paired Mann–Whitney U test, *p* < 1e^−4^). This indicates that gene regulatory links with increased confidence show an even more pronounced recombination rate valley.

We repeated this analysis using physical chromosomal interactions defined by chromatin interaction analysis using paired-end tag sequencing (ChIA-PET), a complementary technique that defines long-range looping interactions in the context of a specific regulator [22]. We used regulatory domains based on ChIA-PET for both polymerase (Pol)II and CTCF as defined by the ENCODE consortium. We found that the recombination rate within ChIA-PET PolII linked regions was also significantly depleted, but not in ChIA-PET CTCF linked regions, which are not gene regulatory domains (Additional file 1: Figure S11k–n).

These results indicate that the recombination rate valley is a general property of gene regulatory domains defined using long-range physical DNA interactions not insulated by CTCF.

### Gene regulatory domains defined using activity correlation show a recombination valley

We next evaluated the relationship between the recombination rate and gene regulatory links defined between enhancer regions and their target genes as predicted using histone modification, DNase accessibility, and gene expression data from the ENCODE [23] and Roadmap Epigenomics Consortia [24]. We used 29,557,079 unique correlation-based links predicted between DNase-seq peaks and gene expression of putative target transcripts (details in Additional file 2: Supplemental method 10). Given the role of CTCF motifs in guiding chromatin loops [20], we focused on 164,409 unique links that were not interrupted by CTCF motifs and thus more likely to lie in the same chromatin loops. We found significantly reduced recombination rate for regions within these enhancer–TSS domains relative to random pairs (two-way paired Mann–Whitney U test and permutation test, *p* < 1e^−4^; Fig. 1d, h, l; Additional file 1: Figure S1d; Additional file 1: Figure S11i, j).

To avoid multiple counts from the same genomic region, we pruned the DNase–TSS links using a similar approach as for the Hi-C links (Additional file 2: Supplemental method 1) and found similar results (Additional file 1: Figure S2e, j, o). We next repeated the analysis using different thresholds for DNase–TSS links (Additional file 1: Figure S5d–h), different genetic maps (Additional file 1: Figure S6d–h), and more stringent matched random intervals in whole genome (Additional file 1: Figure S7d, h, l, p, t, x, ab), non-coding bases, and intergenic regions (Additional file 1: Figure S8d, h, l), and consistently found significant recombination rate valleys within DNase–TSS links.

We performed an additional analysis with 1,427,744 unique enhancer–TSS links predicted using a modified version of a previously published strategy [23] based on cell type-specific chromatin state assignments and correlation between multiple histone modifications and gene expression levels across cell types [24] (details in Additional file 2: Supplementary methods 11). We found that the resulting 139,043 gene regulatory domains without CTCF motifs continued to show a significant depletion in recombination rate at both intermediate and long distances (Additional file 1: Figure S11a–d).

We repeated this analysis using 302,538 unique links predicted using a module-based joint latent Dirichlet allocation (joint-LDA) linking approach (Wang et al., in preparation; the links of data and code are available in Additional file 3: Table S1) that does not depend on correlation and can predict cell type-specific links. Despite these differences in predicting enhancer–TSS links, we found a similar depletion in the recombination rate within gene regulatory domains compared to random controls (Additional file 1: Figure S11e–h).

Together, these results indicate that gene regulatory domains defined based on functional genomics and epigenomic information are associated with a recombination rate valley, indicating that genes tend to be co-inherited with their gene regulatory elements.

### Constitutive and developmental domains show stronger recombination rate depletion

**Fig. 2:**
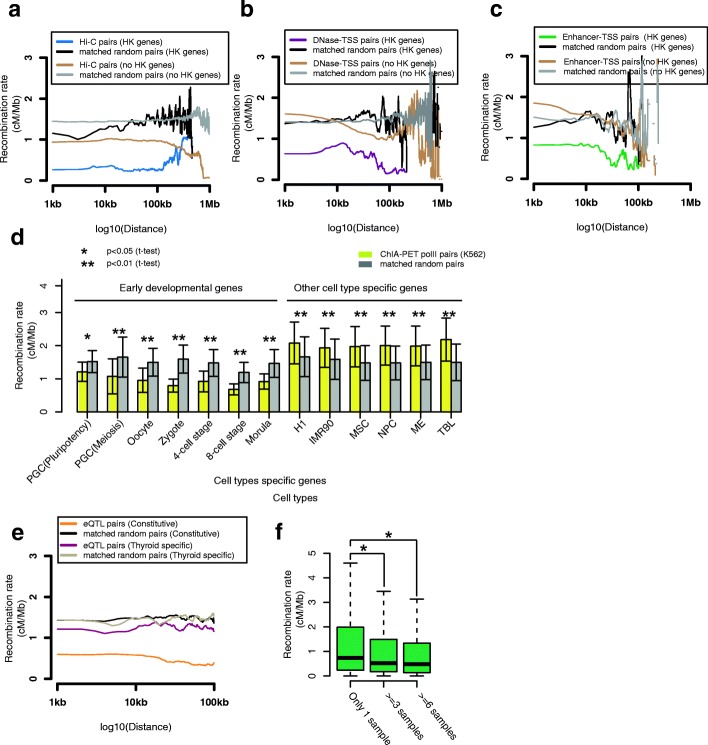
Recombination rate valleys are most prominent at functional links associated with housekeeping genes and constitutive links. **a** Average recombination rate in the top 10% of Hi-C pairs (observed/expected (O/E), no CTCF), associated with housekeeping genes (*blue*) and not associated with housekeeping genes (*golden*). **b** Average recombination rate in DNase–TSS pairs without intervening CTCF motifs, associated with housekeeping genes (*purple*) and not associated with housekeeping genes (*golden*). **c** Average recombination rate in enhancer–TSS pairs called by the LDA method without intervening CTCF motifs, associated with housekeeping genes (*green*) and not associated with housekeeping genes (*golden*). **d** Average recombination rate in ChIA-PET PolII links at early embryonic development genes and other cell type-specific genes in K562 cells. Error bars indicate the standard deviation. **e** The recombination rate valley is much more significant at constitutive eQTL links (*orange*) than that at tissue-specific eQTL links (*magenta*). **f** Recombination rate within enhancer–TSS links (10–100 kb region) called by joint LDA method in different numbers of cell types. Error bars indicate the standard deviation

We next evaluated how the strength of the recombination rate valley varies for different classes of genes. We found that the recombination rate valley within physical and activity links was more pronounced for housekeeping genes [25] compared with non-housekeeping genes (Fig. 2a–c; Additional file 1: Figure S13). It was also more pronounced for genes that act in early embryonic development stages [26, 27], especially for genes in the oocyte stage and genes responsible for meiosis in the primordial germ cell (PGC), but not for most of the other cell type-specific gene groups [28] (Fig. 2d; Additional file 1: Figure S14). To exclude the possibility that the signal in housekeeping genes is due to the contribution of genes actively expressed in oocyte and the early developmental stage and thus not amenable to recombination, we split housekeeping genes into three categories: those highly expressed in early developmental stages (top 10% of expression levels), those not in the top 10%, and those not in the top 50%. Recombination rate valleys were observed no matter the expression levels (Additional file 1: Figure S15).

We also evaluated how the strength of the recombination rate valley varies for different classes of regulatory elements. We evaluated recombination rate depletion using 43,236 constitutive eQTLs and 18,879 thyroid-specific eQTL links from the GTEx project [15]. We found that constitutive eQTL links showed consistently larger discrepancies in recombination rates than tissue-specific links, each compared to matched random controls (Fig. 2e; Additional file 1: Figure S16). Similarly, we found that gene regulatory domains recovered independently in multiple cell types showed a more pronounced recombination rate valley than tissue-specific gene regulatory domains (Fig. 2f).

Thus, the recombination rate valley is more strongly pronounced in gene regulatory domains of constitutively expressed genes, genes with developmental roles, and regulatory elements with constitutive activity, which all share the feature that they are under stronger evolutionary constraint [29]. This suggests that a reduced recombination rate between regulatory elements and their target genes may be advantageous for genes and regulatory elements under stronger selection in the germ line lineage, possibly by facilitating maintenance of the paired gene and its regulatory elements in each allele, which are important during early development, as a single unit of inheritance.

### Recombination rate valleys in mice

We reasoned that if the recombination rate valley is a selected feature of gene regulatory domains in human, it should be an evolutionarily conserved feature in other mammals. To test this hypothesis, we repeated our analysis in the mouse genome.

We quantified recombination rates across the mouse genome using the *Mus musculus* genetic map [30]. We defined gene regulatory domains using both genetic and physical interactions. For genetic interactions, we used 2659 eQTLs based on 100 strains in murine liver [31] and 1035 eQTLs based on 39 strains in two murine immunological cell types [32]. For physical interactions, we used 271,236 Hi-C links (no CTCF) [20] called in a murine lymphoblastoid cell line.

**Fig. 3:**
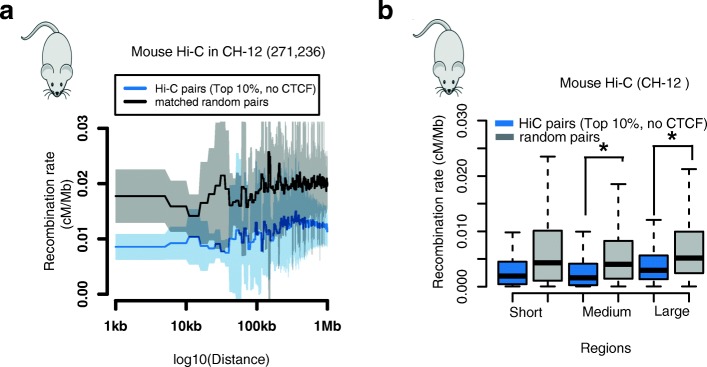
Recombination rate valleys in mouse regulatory domains. **a** Average recombination rate in the top 10% of Hi-C links (observed/expected (O/E)) without intervening CTCF peaks in CH-12 cells. **b** Recombination rate in the top 10% of Hi-C links (O/E) without intervening CTCF peaks in CH-12 cells. Asterisks represent it is statistically significant different between two groups (two-way paired Mann–Whitney U test, *p* < 1e−4)

Evaluating the recombination rate of gene regulatory domains, we found a significant depletion in the recombination rate relative to random pairs for both long genetic interactions and physical interactions (two-way paired Mann–Whitney U test and permutation test, *p* < 1e^−4^; Fig. 3; Additional file 1: Figure S17). We did not observe recombination rate valleys in intermediate-range intervals in genetic links, likely due to the longer LD structure and much lower resolution genetic maps in mouse. This suggests that the recombination rate valley is not a feature solely of the human genome, but may represent a more general mammalian property, possibly as an evolutionarily conserved mechanism to preserve important regulatory domains.

### Potential roles of DNA methylation and double-stranded breaks in recombination rate valleys

**Fig. 4:**
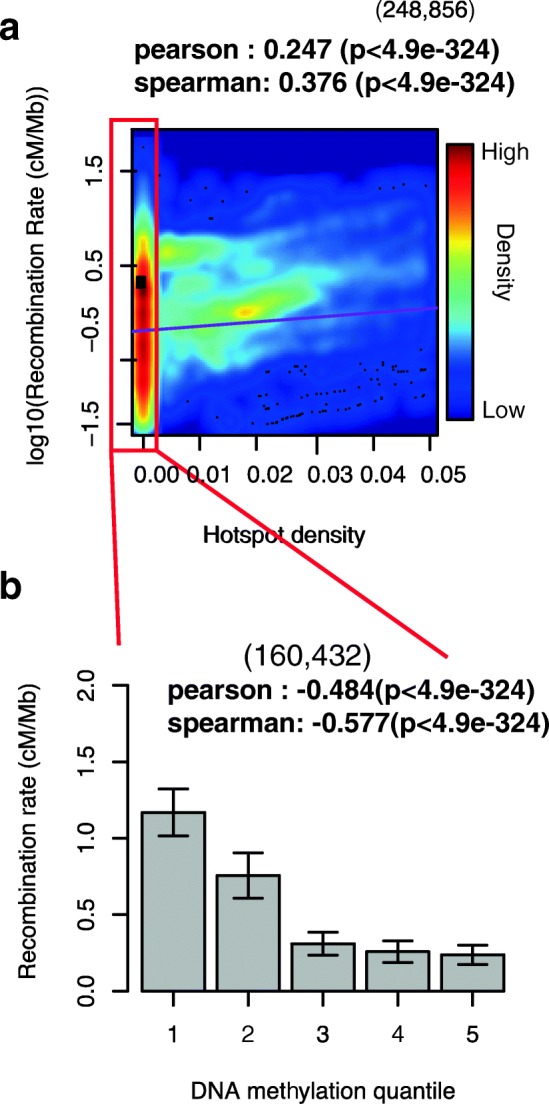
Recombination rate valleys are correlated with hotspot density and DNA methylation. **a** The relationship between log10(recombination rate) and recombination hotspot density (per kb) at each eQTL interval. *Heat colors* represent the point density. Recombination hotspot density less than 0.005/kb (*red rectangle*) was extracted for the analysis in **b. b** The relationship between average recombination rate and DNA methylation quantiles in GV oocyte stage within eQTL intervals. *Error bars* indicate the standard deviation

We next sought to understand potential mechanistic processes that could lead to the observed recombination rate valleys. We found that a scarcity of recombination hotspots is associated with recombination rate valleys (Fig. 4a; Additional file 1: Figure S18a). However, half of links do not have recombination hotspots, and thus their recombination rate variation must be explained using other mechanisms.

Given the previously proposed roles of DNA methylation in recombination rate [11], we studied the relationship between DNA methylation and recombination rate valleys. We used nucleotide-resolution genome-wide methylation profiles in human primordial germ cells (PGCs) [27] and oocytes [33], representing the methylome state of human cells both before and during meiotic arrest (Additional file 1: Figure S19a), in which recombination occurs via crossover events.

We used 500-kb non-overlapping windows to scan the genome and found a strong global negative correlation between methylation levels in PGCs and recombination rate (Additional file 1: Figure S19b; Additional file 1: Figure S20a), indicating that DNA methylation levels immediately prior to recombination events are highly predictive of recombination valleys. In contrast, we did not find such a strong global anti-correlation in oocytes (Additional file 1: Figure S19b); however, methylation level within genetic links such as eQTL links showed negative correlation with recombination rate in oocytes (Fig. 4b; Additional file 1: Figure S18b; Additional file 1: Figure S19c; Additional file 1: Figure S20b–d). These results suggest that DNA methylation may play a role in reducing the frequency of meiotic recombination events that impair paternal and maternal functional regulatory links. We did not find strong global or local negative correlations between methylation and recombination rate in additional cell types in a number of developmental stages (Additional file 1: Figure S19c; Additional file 1: Figure S20b–d).

Recombination events are initiated by double-stranded breaks, which are suggested to be associated with DNA methylation [34]. Thus, methylation of the DNA in large regulatory domains may explain the reduced recombination rate. To evaluate this model, we examined the correlation between DNA methylation levels and double strand break (DSB) initiation frequency, both profiled in sperm cells. We found that DNA methylation showed a significant negative correlation with DSB initiation frequency (Pearson −0.11, *p* value = 1.71e^−16^; Additional file 1: Figure S21a). To further investigate the relationship between DNA methylation and DNA DSBs, we correlated DNA methylation levels profiled in LCL with ChIP-Seq evidence for gamma-H2A.X, markers of double-stranded break repair and active form of H2A.X, profiled in CD4+ T cells [35, 36], and found a significant positive correlation with evidence of DNA repair (Pearson 0.36 with gamma-H2A.X, *p* value < 10^−100^, Additional file 1: Figure S21b). We found a negative correlation of DNA methylation with H2A.X (Pearson −0.211, *p* value = 7.92e^−59^), ruling out the possibility that the association between DNA methylation and gamma-H2A.X is due to the background H2A.X level (Additional file 1: Figure S21c). These results suggest the increased level of DNA methylation in recombination rate valleys may reduce the frequency of DSB initiation and increase the rate of DSB repair, thus contributing to a reduced recombination rate (Additional file 1: Figure S19d).

To quantify the variation in recombination rate due to DNA methylation levels, and regulatory domains defined by genetic, physical, and activity links, we built a random forest regression model that utilizes DNA methylation and regulatory links as features to predict the recombination rate in a genomic interval. The model included adjustments for the varying recombination rate of different chromosomes and at different genomic distances (Additional file 2: Supplementary methods 12 and 13).

**Fig. 5:**
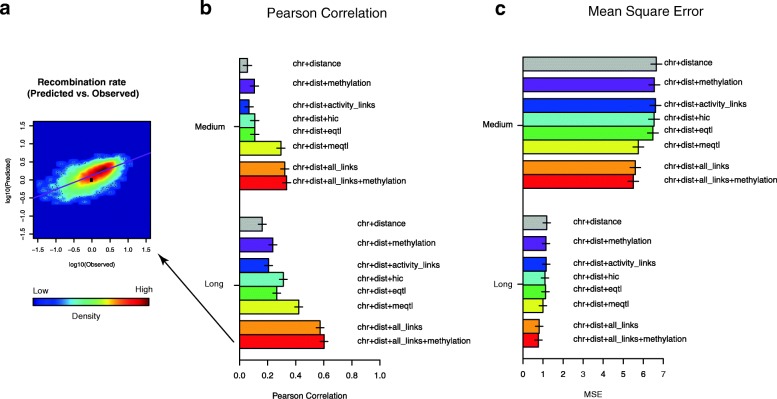
Recombination rate predictions within random intervals. **a** Predicted recombination rate vs. observed recombination rate within random intervals (100 kb–1 Mb region, using chromosome, genomic distance, overlapped fraction with all functional links, and DNA methylation level). The *purple line* represents the fitted linear relationship between the two variables. **b** Average Pearson correlation coefficient between predicted recombination rate and observed recombination rate in medium (10–100 kb) and long (100 kb–1 Mb) distance regions. **c** Average mean squared error between predicted recombination rate and observed recombination rate in medium (10–100 kb) and long (100–1 Mb) distance regions

We found that the individual link types vary greatly in predictive power, with meQTLs showing the strongest predictive power for both medium-range and long-range links. The combination of chromosome number, physical distance, regulatory links, and DNA methylation level resulted in high concordance between predicted and observed recombination rate (Pearson correlation coefficient 0.622, *p* value < 10^−100^). Interestingly, the most accurate predictor used a combination of all four link types and DNA methylation (Fig. 5). In addition, a combined predictor using recombination hotspots and DNA methylation jointly could recapitulate up to 92% of the observed recombination rate difference at long distance and 80% at medium distance within each type of functional link (Additional file 1: Figure S22).

## Discussion

Human recombination rates vary across the genome due to both evolutionary and molecular processes. It is well established that recombination rates are depleted within gene bodies, consistent with their functional role [5, 6]. In this study, we found “recombination rate valleys” of reduced recombination rates between regulatory elements and their target genes defined using a multitude of methods: genetic links (eQTLs and meQTLs), physical links (Hi-C and ChIA-PET), and activity links (enhancer–gene pairs based on histone modification, DNase accessibility, and gene expression).

Our findings suggest that regulatory elements and their target genes form larger functional units. Further supporting this hypothesis, we found recombination rate valleys in Hi-C and DNase–TSS links not interrupted by a CTCF motif, but not links overlapping a CTCF motif. Similarly, we found recombination rate valleys in ChIA-PET PollI interactions, but not ChIA-PET CTCF interactions. We found recombination rate valleys between intergenic eQTL/meQTLs and their target genes, suggesting they are not explained by proximity to gene bodies. We also found recombination rate valleys were enriched in housekeeping genes regardless of expression, suggesting they are not explained by active transcription preventing recombination. Links found in multiple tissue types also showed a greater degree of recombination rate valleys than links only in a single or a few tissues.

Fine-scale recombination maps across species have revealed that recombination hotspots are evolutionarily short-lived, but global patterns of recombination rate are relatively conserved [9]. DNA methylation plays a casual repressive role in meiotic recombination [13, 14, 37, 38], and distantly related species have similar global DNA methylation patterns [39], suggesting that DNA methylation could potentially drive global variation in recombination rate. Supporting this hypothesis, we found a strong global negative correlation between methylation levels in PGCs and recombination which we did not find in oocytes. However, we did find recombination rate valleys in oocytes, suggesting that global DNA demethylation allows recombination in PGCs, but methylation of regulatory links in oocytes might establish recombination rate valleys by preventing double stranded breaks and enhancing DNA repair. Recent genetic variation could also affect DNA methylation, which in turn could drive local variation in recombination rates [10].

Together, our results establish the existence of depleted recombination rates between regulatory elements and their target genes and suggest a mechanistic model involving DNA methylation at the crossover stage. Further work is needed to check if specific combinations of regulatory and genic alleles are under selection and preserved by reduced recombination rates between regulatory elements and their target genes. However, recombination rate valleys might instead be explained by depletion of PRDM9 motifs in the regulatory domains, or by inaccessible chromatin during the crossover across the regulatory links.

## Conclusions

Our results indicate the existence of a recombination rate valley at regulatory domains, consisting of regulatory elements and their target genes. DNA methylation can explain both local and global variations in recombination rate, providing a model to interpret the relationship between genetic and epigenetic variation across individuals.

## Methods

### Generating matched random genomic intervals by iterations

For each functional link, one random interval with the same exact physical length in the same chromosome was generated by Java script “RandomMatchedInterval.java”. The Java script could also produce more stringent matched random intervals with matched GC percentage, CpG density, SNP density, and PRDM9 motif density. For each functional link, RandomMatchedInterval.java will repeatedly generate one random interval with the same length in the same chromosome and calculate the Euclidean distance of these additional features between this function link and the newly generated random interval. It will stop when the Euclidean distance between the functional link and random interval is less than 0.01 or the algorithm reaches the maximum iterations (10,000 iterations). Random intervals with the exact same physical length in the same HiCCUPS domain [20] were generated by “generate_random_in_tads.pl” scripts. A two-way paired Mann–Whitney U test was used to test the significance level between functional links and matched random pairs. *P* value less than 1e^−4^ was used as the significance level threshold.

### Generating matched genomic intervals by k-d tree

In order to obtain more meaningful matched control intervals, we also generated more stringent matched intervals by using a k-dimensional tree (k-d tree) algorithm implemented in “RandomMatchedIntervalByKdtreeByChr.java”. We first generated intervals with all possible lengths (length increases from 1 kb to 1 Mb with 1-kb incremental steps, resulting in 3,083,677,136 intervals) in the human genome (hg19). We retrieved the chromosome number, interval length, GC percentage, CpG density, SNP density, PRDM9 motif density, gene density (for genetic intervals, and distance to the nearest transcription start site (TSS)) for each interval. Then we built a k-d tree for these intervals. For each functional link, we searched its nearest 1000 neighbors in this k-d tree. We filtered the matched intervals when their distance was less than 1 kb away or more than 50% overlapped with the original functional links to avoid sampling the same position again. For physical links and activity links not overlapped with CTCF motifs, we filtered out random links that overlapped with CTCF motifs. Finally, we randomly chose one of these nearest neighbors. Due to the large data size, we generated k-d tree data structure and processed functional links chromosome by chromosome.

### Generating random pairs by bootstrapping

The null distribution of median recombination rate within eQTL pairs was created by the following steps. 1) All possible pairs between SNPs in the genotyping array and TSS in the genome within three distance ranges of the SNP were generated (short, 1–10 kb; medium, 10–100 kb; long, 100 kb–1 Mb). 2) For each of the three genomic intervals, the same number of random pairs within the same genomic distance range as eQTL pairs were randomly sampled. The median recombination value from sampled random pairs was calculated. 3) Step 2 was repeated 10,000 times and the null distribution of median recombination rate within each of these three genomic intervals was obtained. 4) The median eQTL pair recombination rate within the three intervals was ranked in comparison to the three null distributions. The permutation *p* value was therefore obtained. Similar steps were applied to meQTL pairs, Hi-C links, DNase–TSS links, enhancer–TSS links, and ChIA-PET links. The detailed method was implemented as “CalPvalueNullDist.java”. *P* value less than 1e^−4^ was used as the significance level threshold.

## Additional files

Additional file 1: Figure S1. Scatter plot of recombination rate within genetic, physical, and activity links. **Figure S2.** Recombination valleys within non-overlapped genetic, physical, and activity links. **Figure S3.** Differences in recombination rate between best meQTL pairs and locally adjacent pairs. **Figure S4.** Recombination valleys in eQTLs in different tissues and cell lines. **Figure S5.** Recombination valleys within functional links at different thresholds. **Figure S6.** Recombination valleys in different recombination rate maps. **Figure S7.** Recombination valleys after controlling for physical length, G + C percentage, CpG density, SNP density, PRDM9 motif frequency, gene density, and distance to TSS. **Figure S8.** Recombination valleys exist in intergenic regions and non-coding bases. **Figure S9.** Recombination rate between Hi-C pairs and matched random intervals within the same HiCCUPS loops. **Figure S10.** eQTL evidence supported by chromatin conformation signals in the same cell line shows stronger depletion of recombination rate. **Figure S11.** Relationship between recombination valleys and CTCF. **Figure S12** Recombination valleys between physical links, activity links without CTCF motifs, and matched random intervals also without CTCF motifs. **Figure S13.** Recombination valleys are most prominent at enhancer–TSS links, DNase–TSS links Hi-C links, and ChIA-PET PolII/PolII links associated with housekeeping genes. **Figure S14.** Recombination valleys are prominent at early embryonic developmental genes, but not at other cell type-specific genes. **Figure S15.** Recombination valleys are prominent at housekeeping genes in highly expressed and minimally expressed genes at the oocyte stage. **Figure S16.** Recombination valleys are most prominent at constitutive eQTL links. **Figure S17.** Recombination valleys in mouse regulatory domains. **Figure S18.** Recombination valleys are correlated with hotspot density and DNA methylation. **Figure S19.** Mechanistic model for recombination valley in regulatory domains. **Figure S20.** Relationship between recombination rate and DNA methylation quantile within 500-kb windows and within genetic links at different early development stages. **Figure S21.** Global relationship between DNA methylation, DNA double stranded break initiation frequency, and DNA double stranded break repair efficiency. **Figure S22** Recombination rate predictions within functional links. (ZIP 46345 kb)

Additional file 2: Supplementary methods. (DOCX 65 kb)

Additional file 3: Table S1. Datasets used in this study. (XLSX 34 kb)

## Abbreviations

ChIA-PET: Chromatin interaction analysis using paired-end tag sequencing
DSB: Double strand break
eQTL: Expression quantitative trait loci
FDR: False discovery rate
gEUVADIS: Genetic European Variation in Health and Disease
GTEx: Gene-Tissue Expression
Hi-C: Chromosome conformation capture
Joint-LDA: Joint linear-discriminant analysis
LD: Linkage disequilibrium
meQTL: Methylation quantitative trait locus
MuTHER: Multiple Tissue Human Expression Resource
PGC: Primordial germ cell
TSS: Transcription start sites
