# Supplementary material for: Evidence of reduced recombination rate in human regulatory domains
Source: Genome Biol. 2017 Oct 20;18:193. doi: 10.1186/s13059-017-1308-x (PMC5651596; doi:10.1186/s13059-017-1308-x)
Supplement: Supplementary file 1 — Scatter plot of recombination rate within genetic, physical, and activity links. Figure S2. Recombination valleys within non-overlapped genetic, physical, and activity links. Figure S3. Differences in recombination rate between best meQTL pairs and locally adjacent pairs. Figure S4. Recombination valleys in eQTLs in different tissues and cell lines. Figure S5. Recombination valleys within functional links at different thresholds. Figure S6. Recombination valleys in different recombination rate maps. Figure S7. Recombination valleys after controlling for physical length, G + C percentage, CpG density, SNP density, PRDM9 motif frequency, gene density, and distance to TSS. Figure S8. Recombination valleys exist in intergenic regions and non-coding bases. Figure S9. Recombination rate between Hi-C pairs and matched random intervals within the same HiCCUPS loops. Figure S10. eQTL evidence supported by chromatin conformation signals in the same cell line shows stronger depletion of recombination rate. Figure S11. Relationship between recombination valleys and CTCF. Figure S12 Recombination valleys between physical links, activity links without CTCF motifs, and matched random intervals also without CTCF motifs. Figure S13. Recombination valleys are most prominent at enhancer–TSS links, DNase–TSS links Hi-C links, and ChIA-PET PolII/PolII links associated with housekeeping genes. Figure S14. Recombination valleys are prominent at early embryonic developmental genes, but not at other cell type-specific genes. Figure S15. Recombination valleys are prominent at housekeeping genes in highly expressed and minimally expressed genes at the oocyte stage. Figure S16. Recombination valleys are most prominent at constitutive eQTL links. Figure S17. Recombination valleys in mouse regulatory domains. Figure S18. Recombination valleys are correlated with hotspot density and DNA methylation. Figure S19. Mechanistic model for recombination valley in regulatory domains. Figure S20. Relation [file 13059_2017_1308_MOESM1_ESM.zip › Additional file 2-Supplementary Figure Legends.docx]

**Supplementary Figures**

Supplementary Figure 1. **Scatter plot of recombination rate within genetic, physical, and activity links**. Heatmap density plot of recombination rate between (a) meQTL pairs, (b) eQTL pairs, (c) the top 10% of Hi-C pairs (Observed/Expected (O/E), no CTCF motif), (d) DNase-TSS pairs (no CTCF motif) and matched random pairs generated by Supplementary Method 1.

Supplementary Figure 2. **Recombination valleys within non-overlapped genetic, physical, and activity links.** After pruning overlapped links, the average recombination rate within (a) meQTL pairs (red), (b) eQTL pairs in whole blood from GTEx (orange), (c) eQTL pairs in LCL from gEUVADIS (orange), (d) the top 10% of Hi-C pairs (Observed/Expectated (O/E), no CTCF motif, blue), (e) DNase-TSS pairs (no CTCF motif, purple) remain significantly lower than matched random intervals. Recombination rate in three different genomic scales in (f) meQTL pairs, (g) eQTL pairs in whole blood from GTEx (orange), (h) eQTL pairs in LCL from gEUVADIS (orange), (i) the top 10% of Hi-C pairs (O/E, no CTCF motif), (j) DNase-TSS pairs (no CTCF motif). Two-way paired Mann-Whitney U tests show that recombination rate within genetic, physical, and activity links at medium and large intervals is significantly less than matched random pairs (p<1e^-4^). Heatmap density plot of recombination rate between (k) meQTL pairs, (l) eQTL pairs in whole blood from GTEx (orange), (m) eQTL pairs in LCL from gEUVADIS (orange), (n) the top 10% of Hi-C pairs (O/E, no CTCF motif), (o) DNase-TSS pairs (no CTCF motif), and matched random pairs.

Supplementary Figure 3. **Differences in recombination rate between best meQTL pairs and locally adjacent pairs**. Best meQTL pairs (smallest FDR value for each CpG) were randomly shifted within +/-2.5kb (red) and +/-5kb (blue) while keeping the original interval lengths. Differences in recombination rate between these best meQTL pairs and their local adjacent pairs are shown as a density plot. A two-way paired Mann-Whitney U test was performed to calculate the p-value.

Supplementary Figure 4. **Recombination valleys in eQTLs in different tissues and cell lines**. Each graph shows the mean recombination rate between genomic features at each interval distance using colored lines, while black lines represent the value in matched random intervals, for varied tissues across multiple eQTL studies, as indicated.

Supplementary Figure 5. **Recombination valleys within functional links at different thresholds**. The recombination rate called by different FDR thresholds within (a) meQTL pairs and (b) eQTL pairs. (c) The recombination rate within Hi-C pairs (no CTCF motif) at different percentage cut-offs (O/E). (d) The recombination rate within DNase-TSS pairs (no CTCF motif) at different percentage cut-offs (correlation). The proportion of links recovered by different FDR thresholds (percentage value) in (e) meQTL pairs (f) eQTL pairs, (g) Hi-C pairs (no CTCF motif), and (h) DNase-TSS pairs (no CTCF motif). The recombination rate called by different FDR thresholds within (a) best, non-overlapped meQTL pairs and (b) best, non-overlapped eQTL pairs

Supplementary Figure 6. **Recombination valleys in different recombination rate maps**. Recombination valleys in the HapMap genetic map within (a) meQTL pairs, (b) eQTL pairs, (c) the top 10% of Hi-C pairs (O/E, no CTCF motif), (d) DNase-TSS pairs (no CTCF motif), (i) best, non-overlapped meQTL pairs and (j) best, non-overlapped eQTL pairs. Recombination valleys in the deCODE genetic map within (e) meQTL pairs, (f) eQTL pairs, (g) the top 10% of Hi-C pairs (O/E, no CTCF motif), (h) DNase-TSS pairs (no CTCF motif), (k) best, non-overlapped meQTL pairs and (l) best, non-overlapped eQTL pairs

Supplementary Figure 7. **Recombination valleys after controlling for physical length, G+C percentage, CpG density, SNP density, PRDM9 motif frequency, gene density, and distance to TSS.** Recombination valleys within (a) meQTL pairs, (b) eQTL pairs, (c) the top 10% of Hi-C pairs (O/E, no CTCF motif) and (d) DNase-TSS pairs (no CTCF motif) after controlling for physical length, G+C percentage, CpG density, SNP density and PRDM9 motif frequency by a rejection sampling approach (Supplementary Method 1). G+C percentage distribution between (e) meQTL pairs, (f) eQTL pairs, (g) the top 10% of Hi-C pairs (O/E, no CTCF motif), (h) DNase-TSS pairs (no CTCF motif) and matched random pairs. CpG density distribution between (i) meQTL pairs, (j) eQTL pairs, (k) the top 10% of Hi-C pairs (O/E, no CTCF motif), (l) DNase-TSS pairs (no CTCF motif) and matched random pairs. SNP density distribution between (m) meQTL pairs, (n) eQTL pairs, (o) the top 10% of Hi-C pairs (O/E, no CTCF motif) (p) DNase-TSS pairs (no CTCF motif) and matched random pairs. PRDM9 motif density distribution between (q) meQTL pairs, (r) eQTL pairs, (s) the top 10% of Hi-C pairs (O/E, no CTCF motif), (t) DNase-TSS pairs (no CTCF motif) and matched random pairs. Recombination valleys within (u) meQTL pairs, (v) eQTL pairs, (w) the top 10% of Hi-C pairs (O/E, no CTCF motif) (x) DNase-TSS pairs (no CTCF motif), (ae) best, non-overlapped meQTL pairs and (af) best, non-overlapped eQTL pairs after controlling for physical length, G+C percentage, CpG density, SNP density, PRDM9 motif frequency, gene density, and distance to TSS (genetic intervals only) by using K-D trees (Supplemental Method 3). Gene density distribution between (y) meQTL pairs, (z) eQTL pairs, (aa) the top 10% of Hi-C pairs (O/E, no CTCF motif), (ab) DNase-TSS pairs (no CTCF motif) and matched random pairs. Position to TSS density distribution between (ac) meQTL pairs, (ad) eQTL pairs and matched random pairs

Supplementary Figure 8. **Recombination valleys exist in intergenic regions and non-coding bases.** The criteria to select intergenic (a) meQTL pairs (b) eQTL pairs (c) the top 10% of Hi-C pairs (O/E, no CTCF motif) and (d) DNase-TSS pairs (no CTCF motif)**.** The recombination rate within intergenic (e) meQTL pairs (f) eQTL pairs (g) the top 10% of Hi-C pairs (O/E, no CTCF motif), (h) DNase-TSS pairs (no CTCF motif), (m) best, non-overlapped meQTL pairs and (n) best, non-overlapped eQTL pairs. Random pairs were matched by chromosome, physical length, G+C percentage, CpG density, SNP density, gene density, and PRDM9 motif frequency. The average recombination rate for only non-coding bases within (i) meQTL pairs (j) eQTL pairs (k) the top 10% of Hi-C pairs (O/E, no CTCF motif), (l) DNase-TSS pairs (no CTCF motif), (o) best, non-overlapped meQTL pairs and (p) best, non-overlapped eQTL pairs.

Supplementary Figure 9. **Recombination rate between Hi-C pairs and matched random intervals within the same HiCCUPS loops.** (a) The average recombination rate between the top 10% of Hi-C pairs (O/E, no CTCF motif, blue) and matched random intervals generated within the same HiCCUPS loops. (b) Recombination rate in three different genomic scales in Hi-C pairs and matched random intervals generated within the same HiCCUPS loops.

Supplementary Figure 10. **eQTL evidence supported by chromatin conformation signals in the same cell line shows stronger depletion of recombination rate**. (a) Recombination rate within cis-eQTL pairs called in the lymphoblastoid cell line (LCL) from the MuTHER project, with no Hi-C signal support, (b) with Hi-C signal support. . (c) Recombination rate within best, non-overlapped cis-eQTL pairs called in the lymphoblastoid cell line (LCL) from the MuTHER project, with no Hi-C signal support, (d) with Hi-C signal support.

Supplementary Figure 11. **Relationship between** **recombination valleys and CTCF**. (a) Average recombination rate in Enhancer-TSS pairs called by the correlation method without intervening CTCF motifs. (b) Recombination rate in Enhancer-TSS pairs called by the correlation method without intervening CTCF motifs. (c) Average recombination rate in Enhancer-TSS pairs called by the correlation method with CTCF motif in-between. (d) Recombination rate in Enhancer-TSS pairs called by the correlation method with CTCF motif in-between. (e) Average recombination rate in Enhancer-TSS pairs called by the LDA method without intervening CTCF motifs. (f) Recombination rate in Enhancer-TSS pairs called by the LDA method without intervening CTCF motifs. (g) Average recombination rate in Enhancer-TSS pairs called by the LDA method with CTCF motif in-between. (h) Recombination rate in Enhancer-TSS pairs called by the LDA method with CTCF motif in-between. (i) Average recombination rate in DNase_peaks-TSS pairs with intervening CTCF motif. (j) Recombination rate in DNase_peaks-TSS pairs with CTCF motif in-between. (k) Average recombination rate in ChIA-PET PolII pairs at K562 cell line (l) Recombination rate in ChIA-PET PolII pairs at K562 cell line. (m) Average recombination rate in ChIA-PET CTCF pairs at K562 cell line (n) Recombination rate in ChIA-PET CTCF pairs at K562 cell line. (o) Average recombination rate in the top 10% of Hi-C links (O/E) with CTCF motif in-between (p) Recombination rate in the top 10% of Hi-C links (O/E) with CTCF motif in-between.

Supplementary Figure 12. **Recombination valleys between physical links, activity links without CTCF motifs, and matched random intervals also without CTCF motifs**. (a) Average recombination rate in top 10% of Hi-C links (O/E), without an intervening CTCF motif. (b) Recombination rate in top 10% of Hi-C links (O/E), without intervening CTCF motifs. (c) Average recombination rate in DNase-TSS pairs called by the correlation method, without CTCF motifs. (d) Recombination rate in DNase-TSS pairs called by the correlation method, without CTCF motifs. All the matched random intervals were generated by supplementary method 3.

Supplementary Figure 13. **Recombination valleys are most prominent at Enhancer-TSS links, DNase-TSS links Hi-C links, and ChIA-PET PolIIPolII links associated with housekeeping genes**. (a) Pie chart showed the percentage of Housekeeping genes (HK gene) in the total number of RefSeq genes (b) Average recombination rates in Enhancer-TSS pairs called by the correlation method without intervening CTCF motifs, associated with housekeeping genes. (c) Recombination rate in Enhancer-TSS pairs called by the correlation method without intervening CTCF motifs, associated with housekeeping genes. (d) Average recombination rate in Enhancer-TSS pairs called by the correlation method without intervening CTCF motifs, not associated with housekeeping genes. (e) Recombination rate in Enhancer-TSS pairs called by the correlation method without intervening CTCF motifs, not associated with housekeeping genes. (f) Average recombination rate in Enhancer-TSS pairs called by the LDA method without intervening CTCF motifs, associated with housekeeping genes. (g) Recombination rate in Enhancer-TSS pairs called by the LDA method without intervening CTCF motifs, associated with housekeeping genes. (h) Average recombination rate in Enhancer-TSS pairs called by the LDA method without intervening CTCF motifs, not associated with housekeeping genes. (i) Recombination rate in Enhancer-TSS pairs called by the LDA method without intervening CTCF motifs, not associated with housekeeping genes. (j) Average recombination rate in DNase-TSS pairs without intervening CTCF motifs, associated with housekeeping genes. (k) Recombination rate in DNase -TSS pairs without intervening CTCF motifs, associated with housekeeping genes. (l) Average recombination rate in DNase-TSS pairs without intervening CTCF motifs, not associated with housekeeping genes. (m) Recombination rate in DNase -TSS pairs without intervening CTCF motifs, not associated with housekeeping genes (n) Average recombination rate in the top 10% of Hi-C pairs (O/E, no CTCF), associated with housekeeping genes (o) Recombination rate in the top 10% of Hi-C pairs (O/E, no CTCF), associated with housekeeping genes. (p) Average recombination rate in the top 10% of Hi-C pairs (O/E, no CTCF), not associated with housekeeping genes (q) Recombination rate in the top 10% of Hi-C pairs (O/E, no CTCF), not associated with housekeeping genes. (r) Average recombination rate in ChIA-PET PolII pairs associated with housekeeping genes at K562 cell line (s) Recombination rate in ChIA-PET PolII pairs associated with housekeeping genes at K562 cell line. (t) Average recombination rate in ChIA-PET PolII pairs not associated with housekeeping genes at K562 cell line (u) Recombination rate in ChIA-PET PolII pairs not associated with housekeeping genes at K562 cell line. (v) Average recombination rate in ChIA-PET PolII pairs associated with housekeeping genes in the MCF7 cell line (w) Recombination rate in ChIA-PET PolII pairs associated with housekeeping genes in the MCF7 cell line. (x) Average recombination rate in ChIA-PET PolII pairs not associated with housekeeping genes in the MCF7 cell line. (y) Recombination rate in ChIA-PET PolII pairs not associated with housekeeping genes in the MCF7 cell line.

Supplementary Figure 14. **Recombination valleys are prominent at early embryonic developmental genes, but not at other cell type specific genes.** (a) Average recombination rate in DNase-TSS pairs (no CTCF) at early embryonic developmental genes and other cell type specific genes. (b) Average recombination rate in Enhancer-TSS pairs (no CTCF) called by the LDA method at early embryonic developmental genes and other cell type specific genes. (c) Average recombination rate in Enhancer-TSS pairs (no CTCF) called by the correlation method at early embryonic developmental genes and other cell type specific genes. (d) Average recombination rate in the top 10% of Hi-C links (O/E, no CTCF) at early embryonic developmental genes and other cell type specific genes. (e) Average recombination rate in ChIA-PET PolII links at early embryonic developmental genes and other cell type specific genes in MCF7 cells.

Supplementary Figure 15. **Recombination valleys are prominent at housekeeping genes in highly expressed and minimally expressed genes at the oocyte stage.** (a) Average recombination rate in the top 10% of Hi-C pairs (O/E, no CTCF) associated with housekeeping genes in the top decile of expression in oocytes. (b) Recombination rate in the top 10% of Hi-C pairs (O/E, no CTCF) associated with housekeeping genes and in the top decile of expression in oocytes. (c) Average recombination rate in the top 10% of Hi-C pairs (O/E, no CTCF) associated with housekeeping genes not in the top decile of expression in oocytes. (d) Recombination rate in the top 10% of Hi-C pairs (O/E, no CTCF) associated with housekeeping genes not in the top decile of expression in oocytes. (e) Average recombination rate in the top 10% of Hi-C pairs (O/E, no CTCF) associated with housekeeping genes not in the top 50% of expression in oocytes. (f) Recombination rate in the top 10% of Hi-C pairs (O/E, no CTCF) associated with housekeeping genes not in the top 50% of expression in oocytes. (g) Average recombination rate in DNase-TSS pairs without intervening CTCF motifs, associated with housekeeping genes in the top decile of expression in oocytes. (h) Recombination rate in DNas-TSS pairs without intervening CTCF motifs, associated with housekeeping genes in the top decile of expression in oocytes. (i) Average recombination rate in DNase-TSS pairs without intervening CTCF motifs, associated with housekeeping genes not in the top decile of expression in oocytes. (j) Recombination rate in DNase-TSS pairs without intervening CTCF motifs, associated with housekeeping genes not in the top decile of expression in oocytes. (k) Average recombination rate in DNase -TSS pairs without intervening CTCF motifs, associated with housekeeping genes not in the top 50% of expression in oocytes. (l) Recombination rate in DNase-TSS pairs without intervening CTCF motifs, associated with housekeeping genes not in the top 50% of expression in oocytes. (m) Average recombination rate in Enhancer -TSS pairs without intervening CTCF motifs, associated with housekeeping genes in the top decile of expression in oocytes. (n) Recombination rate in Enhancer-TSS pairs without intervening CTCF motifs, associated with housekeeping genes in the top decile of expression in oocytes. (o) Average recombination rate in top Enhancer -TSS pairs without intervening CTCF motifs, associated with housekeeping genes not in the top decile of expression in oocytes. (p) Recombination rate in Enhancer-TSS pairs without intervening CTCF motifs, associated with housekeeping genes not in the top decile of expression in oocytes. (q) Average recombination rate in Enhancer -TSS pairs without intervening CTCF motifs, associated with housekeeping genes not in the top 50% of expression in oocytes. (r) Recombination rate in Enhancer-TSS pairs without intervening CTCF motifs, associated with housekeeping genes not in the top 50% of expression in oocytes.

Supplementary Figure 16. **Recombination valleys are most prominent at constitutive eQTL links**. Recombination valley is much more significant at constitutive eQTL links (orange) than that at tissue specific eQTL links (magenta). Only best, non-overlapped links are plotted.

Supplementary Figure 17. **Recombination valleys in mouse regulatory domains.** (a) Average recombination rate in the top 10% of Hi-C links (O/E) with intervening CTCF peaks in CH-12 cells. (b) Average recombination rate in the top 10% of Hi-C links (O/E) with intervening CTCF motifs in CH-12 cells. (c) Average recombination rate in the top 10% of Hi-C links (O/E) without intervening CTCF motifs in CH-12 cells. (d) Recombination rates in the top 10% of Hi-C links (O/E) without intervening CTCF motifs in CH-12 cells. (e) Recombination rate in eQTL pairs in liver cells, (f) jointly called in mouse CD4+ T cell and granulocytes. (g) called specifically at either CD4+ T cell or granulocytes in mouse. (h) Recombination rate in best, non-overlapped eQTL pairs in liver cells, (i) jointly called in mouse CD4+ T cell and granulocytes. (j) called specifically at either CD4+ T cell or granulocytes in mouse.

Supplementary Figure 18. **Recombination valleys are correlated with hotspot density and DNA methylation**. (a) The relationship between log10(recombination rate) and recombination hotspot density (per kb) at each eQTL interval. Heat colors represent the point density. Recombination hotspot density less than 0.005/kb (red rectangle) was extracted for the analysis in (b). (b) The relationship between average recombination rate and DNA methylation quantiles in GV oocyte stage within eQTL intervals. Error bars indicate the standard deviation. Only best, non-overlapped links are plotted

Supplementary Figure 19. **Mechanistic model for recombination valley in regulatory domains**. (a) Germ cell lineage in male and female. (b) Pearson correlation coefficient between recombination rate and DNA methylation quantiles within 500kb non-overlapped windows at different early development stages. (c) Pearson correlation coefficient between recombination rate and DNA methylation quantiles within eQTL pairs at different early development stages. (d) The model of recombination valley formation at regions with and without recombination hotspots.

Supplementary Figure 20. **Relationship between recombination rate and DNA methylation quantile within 500kb window and within genetic links at different early development stages**. (a) Barplot of the Spearman correlation coefficient between recombination rate and DNA methylation quantiles within 500kb non-overlapped windows at early developmental stages. (b) Barplot of the Spearman correlation coefficient between recombination rate and DNA methylation quantiles within eQTL pairs at early developmental stages. Barplot of the (c) pearson and (d) spearman correlation coefficient between recombination rate and DNA methylation quantiles within best, non-overlapped eQTL pairs at early developmental stages

Supplementary Figure 21. **Global relationship between** **DNA methylation, DNA double stranded break initiation frequency, and DNA double stranded break repair efficiency**. Normalized (a) DNA double stranded break (DSB) initiation, (b) gamma H2A.X and (c) H2A.X z-score in 500kb non-overlapping windows at each DNA methylation quantile.

Supplementary Figure 22. **Recombination rate predictions within functional links**. Barplot of the average Pearson correlation coefficient between predicted recombination rate and observed recombination rate in medium (10kb-100kb) and long distance (100kb-1Mb) regions at (a) meQTL pairs (d) eQTL pairs (g) Hi-C links (top 10% O/E, no CTCF) (j) activity links (DNase-TSS links, no CTCF). Barplot of the average Spearman correlation coefficient between predicted recombination rate and observed recombination rate quantiles in medium (10kb-100kb) and long distance (100kb-1Mb) regions at (b) meQTL pairs (e) eQTL pairs (h) Hi-C links (top 10% O/E, no CTCF) (k) activity links (DNase-TSS links, no CTCF). Barplot of the average mean square error (MSE) between predicted recombination rate and observed recombination rate in medium (10kb-100kb) and long distance (100kb-1Mb) regions at (c) meQTL pairs (f) eQTL pairs (i) Hi-C links (top 10% O/E, no CTCF) (l) activity links (DNase-TSS links, no CTCF).
