# Supplementary figures and images for: Evidence of reduced recombination rate in human regulatory domains
Source: Genome Biol. 2017 Oct 20;18:193. doi: 10.1186/s13059-017-1308-x (PMC5651596; doi:10.1186/s13059-017-1308-x)

# Supplementary Figure 1

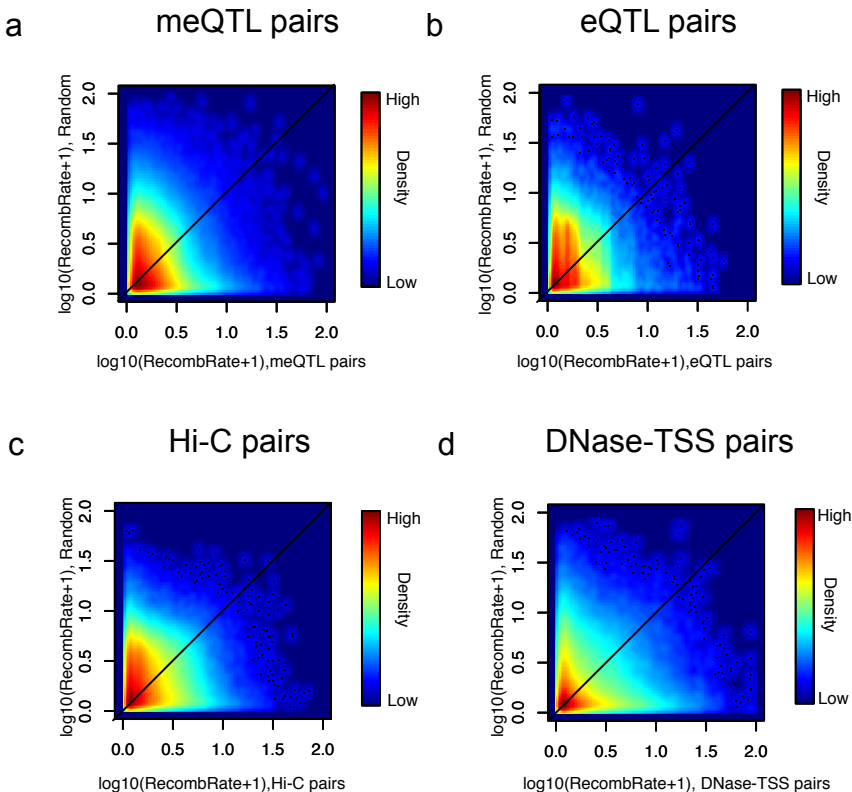

Supplement: Supplementary file 1 — Scatter plot of recombination rate within genetic, physical, and activity links. Figure S2. Recombination valleys within non-overlapped genetic, physical, and activity links. Figure S3. Differences in recombination rate between best meQTL pairs and locally adjacent pairs. Figure S4. Recombination valleys in eQTLs in different tissues and cell lines. Figure S5. Recombination valleys within functional links at different thresholds. Figure S6. Recombination valleys in different recombination rate maps. Figure S7. Recombination valleys after controlling for physical length, G + C percentage, CpG density, SNP density, PRDM9 motif frequency, gene density, and distance to TSS. Figure S8. Recombination valleys exist in intergenic regions and non-coding bases. Figure S9. Recombination rate between Hi-C pairs and matched random intervals within the same HiCCUPS loops. Figure S10. eQTL evidence supported by chromatin conformation signals in the same cell line shows stronger depletion of recombination rate. Figure S11. Relationship between recombination valleys and CTCF. Figure S12 Recombination valleys between physical links, activity links without CTCF motifs, and matched random intervals also without CTCF motifs. Figure S13. Recombination valleys are most prominent at enhancer–TSS links, DNase–TSS links Hi-C links, and ChIA-PET PolII/PolII links associated with housekeeping genes. Figure S14. Recombination valleys are prominent at early embryonic developmental genes, but not at other cell type-specific genes. Figure S15. Recombination valleys are prominent at housekeeping genes in highly expressed and minimally expressed genes at the oocyte stage. Figure S16. Recombination valleys are most prominent at constitutive eQTL links. Figure S17. Recombination valleys in mouse regulatory domains. Figure S18. Recombination valleys are correlated with hotspot density and DNA methylation. Figure S19. Mechanistic model for recombination valley in regulatory domains. Figure S20. Relation [file 13059_2017_1308_MOESM1_ESM.zip › Supplemental_Fig_S1.pdf]

# Supplementary Figure 12

Hi-C pairs

DNase-TSS pairs

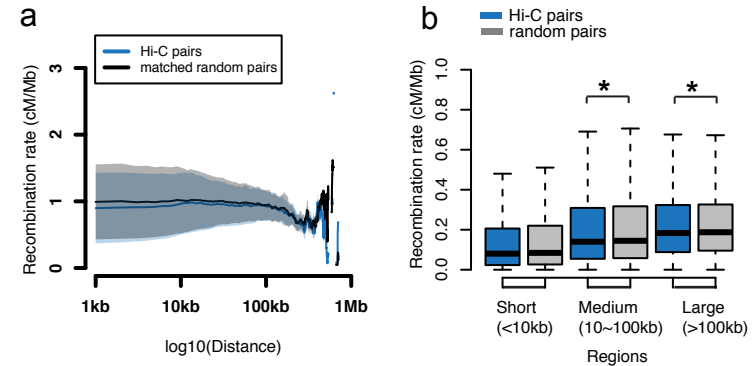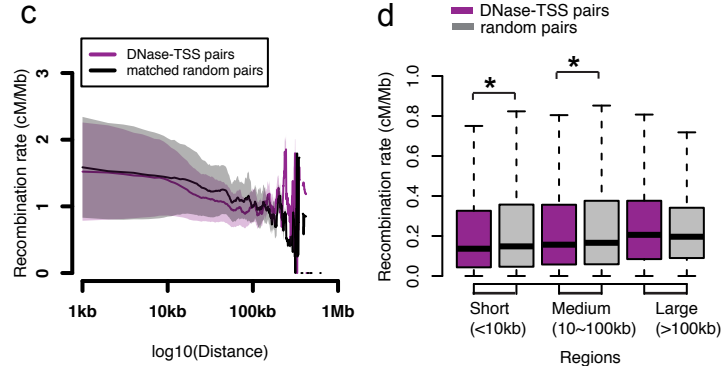

Supplement: Supplementary file 1 — Scatter plot of recombination rate within genetic, physical, and activity links. Figure S2. Recombination valleys within non-overlapped genetic, physical, and activity links. Figure S3. Differences in recombination rate between best meQTL pairs and locally adjacent pairs. Figure S4. Recombination valleys in eQTLs in different tissues and cell lines. Figure S5. Recombination valleys within functional links at different thresholds. Figure S6. Recombination valleys in different recombination rate maps. Figure S7. Recombination valleys after controlling for physical length, G + C percentage, CpG density, SNP density, PRDM9 motif frequency, gene density, and distance to TSS. Figure S8. Recombination valleys exist in intergenic regions and non-coding bases. Figure S9. Recombination rate between Hi-C pairs and matched random intervals within the same HiCCUPS loops. Figure S10. eQTL evidence supported by chromatin conformation signals in the same cell line shows stronger depletion of recombination rate. Figure S11. Relationship between recombination valleys and CTCF. Figure S12 Recombination valleys between physical links, activity links without CTCF motifs, and matched random intervals also without CTCF motifs. Figure S13. Recombination valleys are most prominent at enhancer–TSS links, DNase–TSS links Hi-C links, and ChIA-PET PolII/PolII links associated with housekeeping genes. Figure S14. Recombination valleys are prominent at early embryonic developmental genes, but not at other cell type-specific genes. Figure S15. Recombination valleys are prominent at housekeeping genes in highly expressed and minimally expressed genes at the oocyte stage. Figure S16. Recombination valleys are most prominent at constitutive eQTL links. Figure S17. Recombination valleys in mouse regulatory domains. Figure S18. Recombination valleys are correlated with hotspot density and DNA methylation. Figure S19. Mechanistic model for recombination valley in regulatory domains. Figure S20. Relation [file 13059_2017_1308_MOESM1_ESM.zip › Supplemental_Fig_S12.pdf]

Supplemental Figure 13

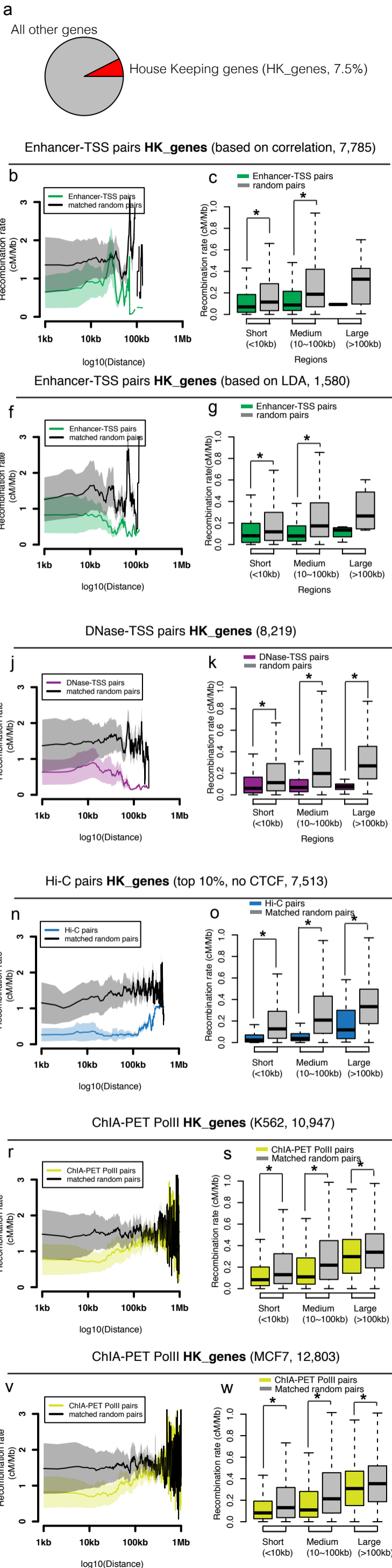

Supplement: Supplementary file 1 — Scatter plot of recombination rate within genetic, physical, and activity links. Figure S2. Recombination valleys within non-overlapped genetic, physical, and activity links. Figure S3. Differences in recombination rate between best meQTL pairs and locally adjacent pairs. Figure S4. Recombination valleys in eQTLs in different tissues and cell lines. Figure S5. Recombination valleys within functional links at different thresholds. Figure S6. Recombination valleys in different recombination rate maps. Figure S7. Recombination valleys after controlling for physical length, G + C percentage, CpG density, SNP density, PRDM9 motif frequency, gene density, and distance to TSS. Figure S8. Recombination valleys exist in intergenic regions and non-coding bases. Figure S9. Recombination rate between Hi-C pairs and matched random intervals within the same HiCCUPS loops. Figure S10. eQTL evidence supported by chromatin conformation signals in the same cell line shows stronger depletion of recombination rate. Figure S11. Relationship between recombination valleys and CTCF. Figure S12 Recombination valleys between physical links, activity links without CTCF motifs, and matched random intervals also without CTCF motifs. Figure S13. Recombination valleys are most prominent at enhancer–TSS links, DNase–TSS links Hi-C links, and ChIA-PET PolII/PolII links associated with housekeeping genes. Figure S14. Recombination valleys are prominent at early embryonic developmental genes, but not at other cell type-specific genes. Figure S15. Recombination valleys are prominent at housekeeping genes in highly expressed and minimally expressed genes at the oocyte stage. Figure S16. Recombination valleys are most prominent at constitutive eQTL links. Figure S17. Recombination valleys in mouse regulatory domains. Figure S18. Recombination valleys are correlated with hotspot density and DNA methylation. Figure S19. Mechanistic model for recombination valley in regulatory domains. Figure S20. Relation [file 13059_2017_1308_MOESM1_ESM.zip › Supplemental_Fig_S13.pdf]

# Supplementary Figure 14

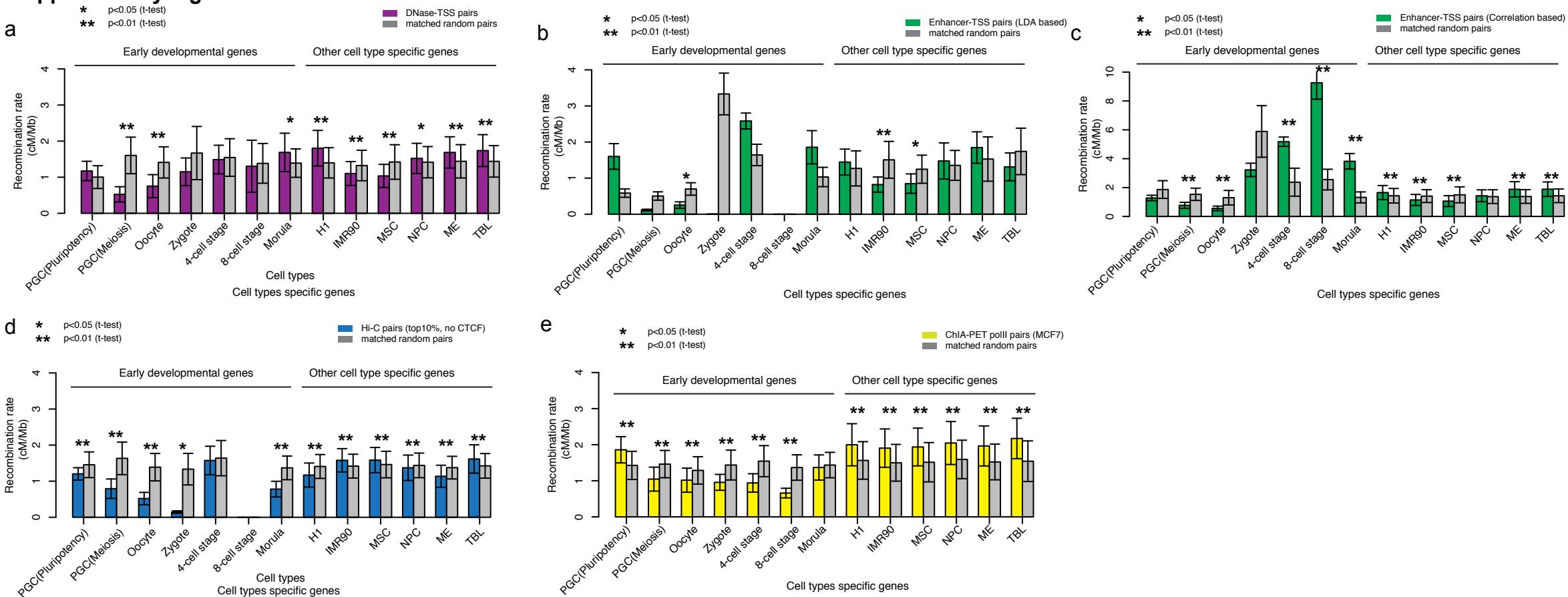

Supplement: Supplementary file 1 — Scatter plot of recombination rate within genetic, physical, and activity links. Figure S2. Recombination valleys within non-overlapped genetic, physical, and activity links. Figure S3. Differences in recombination rate between best meQTL pairs and locally adjacent pairs. Figure S4. Recombination valleys in eQTLs in different tissues and cell lines. Figure S5. Recombination valleys within functional links at different thresholds. Figure S6. Recombination valleys in different recombination rate maps. Figure S7. Recombination valleys after controlling for physical length, G + C percentage, CpG density, SNP density, PRDM9 motif frequency, gene density, and distance to TSS. Figure S8. Recombination valleys exist in intergenic regions and non-coding bases. Figure S9. Recombination rate between Hi-C pairs and matched random intervals within the same HiCCUPS loops. Figure S10. eQTL evidence supported by chromatin conformation signals in the same cell line shows stronger depletion of recombination rate. Figure S11. Relationship between recombination valleys and CTCF. Figure S12 Recombination valleys between physical links, activity links without CTCF motifs, and matched random intervals also without CTCF motifs. Figure S13. Recombination valleys are most prominent at enhancer–TSS links, DNase–TSS links Hi-C links, and ChIA-PET PolII/PolII links associated with housekeeping genes. Figure S14. Recombination valleys are prominent at early embryonic developmental genes, but not at other cell type-specific genes. Figure S15. Recombination valleys are prominent at housekeeping genes in highly expressed and minimally expressed genes at the oocyte stage. Figure S16. Recombination valleys are most prominent at constitutive eQTL links. Figure S17. Recombination valleys in mouse regulatory domains. Figure S18. Recombination valleys are correlated with hotspot density and DNA methylation. Figure S19. Mechanistic model for recombination valley in regulatory domains. Figure S20. Relation [file 13059_2017_1308_MOESM1_ESM.zip › Supplemental_Fig_S14.pdf]

Supplementary Figure 15

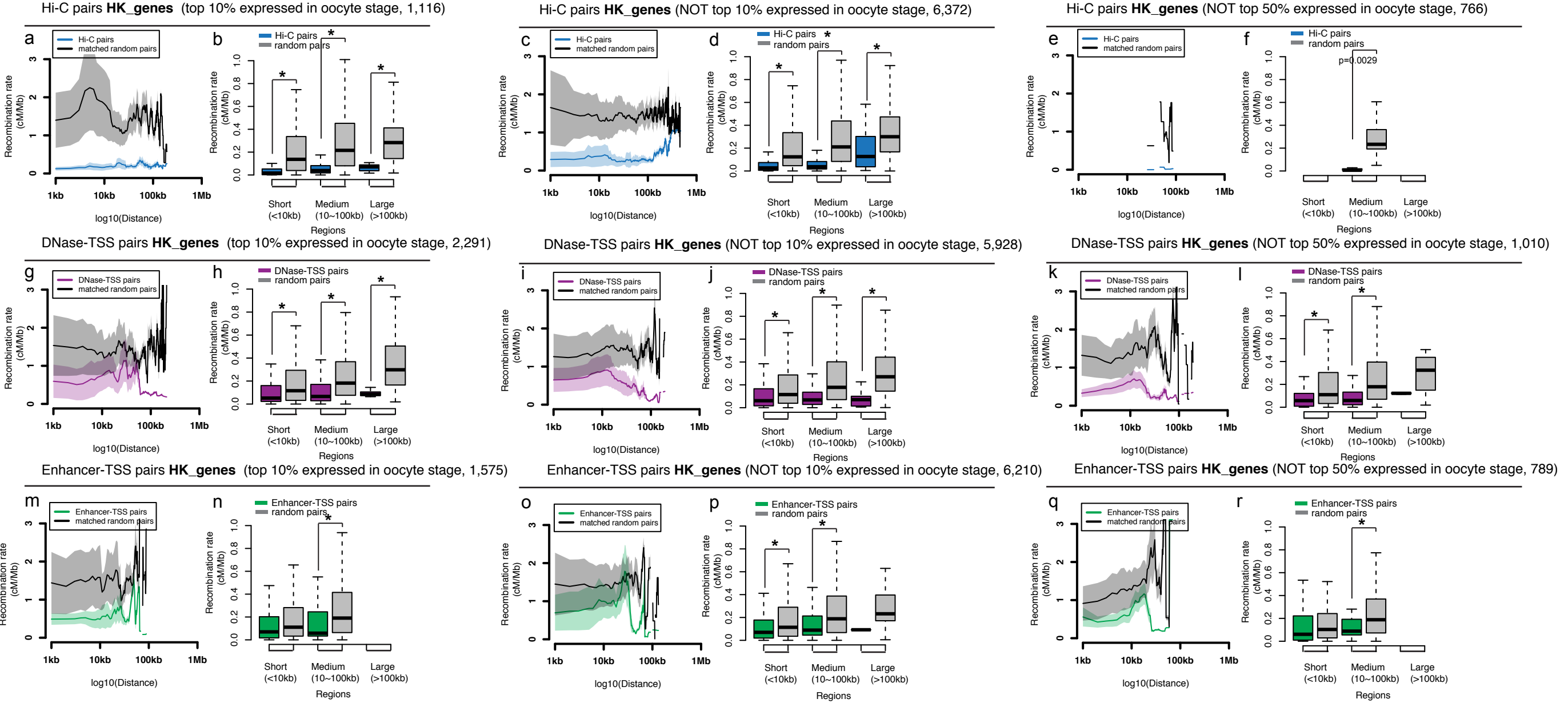

Supplement: Supplementary file 1 — Scatter plot of recombination rate within genetic, physical, and activity links. Figure S2. Recombination valleys within non-overlapped genetic, physical, and activity links. Figure S3. Differences in recombination rate between best meQTL pairs and locally adjacent pairs. Figure S4. Recombination valleys in eQTLs in different tissues and cell lines. Figure S5. Recombination valleys within functional links at different thresholds. Figure S6. Recombination valleys in different recombination rate maps. Figure S7. Recombination valleys after controlling for physical length, G + C percentage, CpG density, SNP density, PRDM9 motif frequency, gene density, and distance to TSS. Figure S8. Recombination valleys exist in intergenic regions and non-coding bases. Figure S9. Recombination rate between Hi-C pairs and matched random intervals within the same HiCCUPS loops. Figure S10. eQTL evidence supported by chromatin conformation signals in the same cell line shows stronger depletion of recombination rate. Figure S11. Relationship between recombination valleys and CTCF. Figure S12 Recombination valleys between physical links, activity links without CTCF motifs, and matched random intervals also without CTCF motifs. Figure S13. Recombination valleys are most prominent at enhancer–TSS links, DNase–TSS links Hi-C links, and ChIA-PET PolII/PolII links associated with housekeeping genes. Figure S14. Recombination valleys are prominent at early embryonic developmental genes, but not at other cell type-specific genes. Figure S15. Recombination valleys are prominent at housekeeping genes in highly expressed and minimally expressed genes at the oocyte stage. Figure S16. Recombination valleys are most prominent at constitutive eQTL links. Figure S17. Recombination valleys in mouse regulatory domains. Figure S18. Recombination valleys are correlated with hotspot density and DNA methylation. Figure S19. Mechanistic model for recombination valley in regulatory domains. Figure S20. Relation [file 13059_2017_1308_MOESM1_ESM.zip › Supplemental_Fig_S15.pdf]

# Supplementary Figure 16

best, non-overlapped eQTL pairs

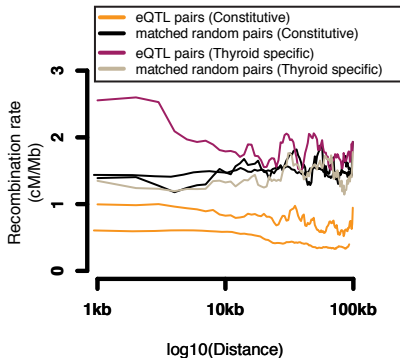

Supplement: Supplementary file 1 — Scatter plot of recombination rate within genetic, physical, and activity links. Figure S2. Recombination valleys within non-overlapped genetic, physical, and activity links. Figure S3. Differences in recombination rate between best meQTL pairs and locally adjacent pairs. Figure S4. Recombination valleys in eQTLs in different tissues and cell lines. Figure S5. Recombination valleys within functional links at different thresholds. Figure S6. Recombination valleys in different recombination rate maps. Figure S7. Recombination valleys after controlling for physical length, G + C percentage, CpG density, SNP density, PRDM9 motif frequency, gene density, and distance to TSS. Figure S8. Recombination valleys exist in intergenic regions and non-coding bases. Figure S9. Recombination rate between Hi-C pairs and matched random intervals within the same HiCCUPS loops. Figure S10. eQTL evidence supported by chromatin conformation signals in the same cell line shows stronger depletion of recombination rate. Figure S11. Relationship between recombination valleys and CTCF. Figure S12 Recombination valleys between physical links, activity links without CTCF motifs, and matched random intervals also without CTCF motifs. Figure S13. Recombination valleys are most prominent at enhancer–TSS links, DNase–TSS links Hi-C links, and ChIA-PET PolII/PolII links associated with housekeeping genes. Figure S14. Recombination valleys are prominent at early embryonic developmental genes, but not at other cell type-specific genes. Figure S15. Recombination valleys are prominent at housekeeping genes in highly expressed and minimally expressed genes at the oocyte stage. Figure S16. Recombination valleys are most prominent at constitutive eQTL links. Figure S17. Recombination valleys in mouse regulatory domains. Figure S18. Recombination valleys are correlated with hotspot density and DNA methylation. Figure S19. Mechanistic model for recombination valley in regulatory domains. Figure S20. Relation [file 13059_2017_1308_MOESM1_ESM.zip › Supplemental_Fig_S16.pdf]

# Supplementary Figure 17

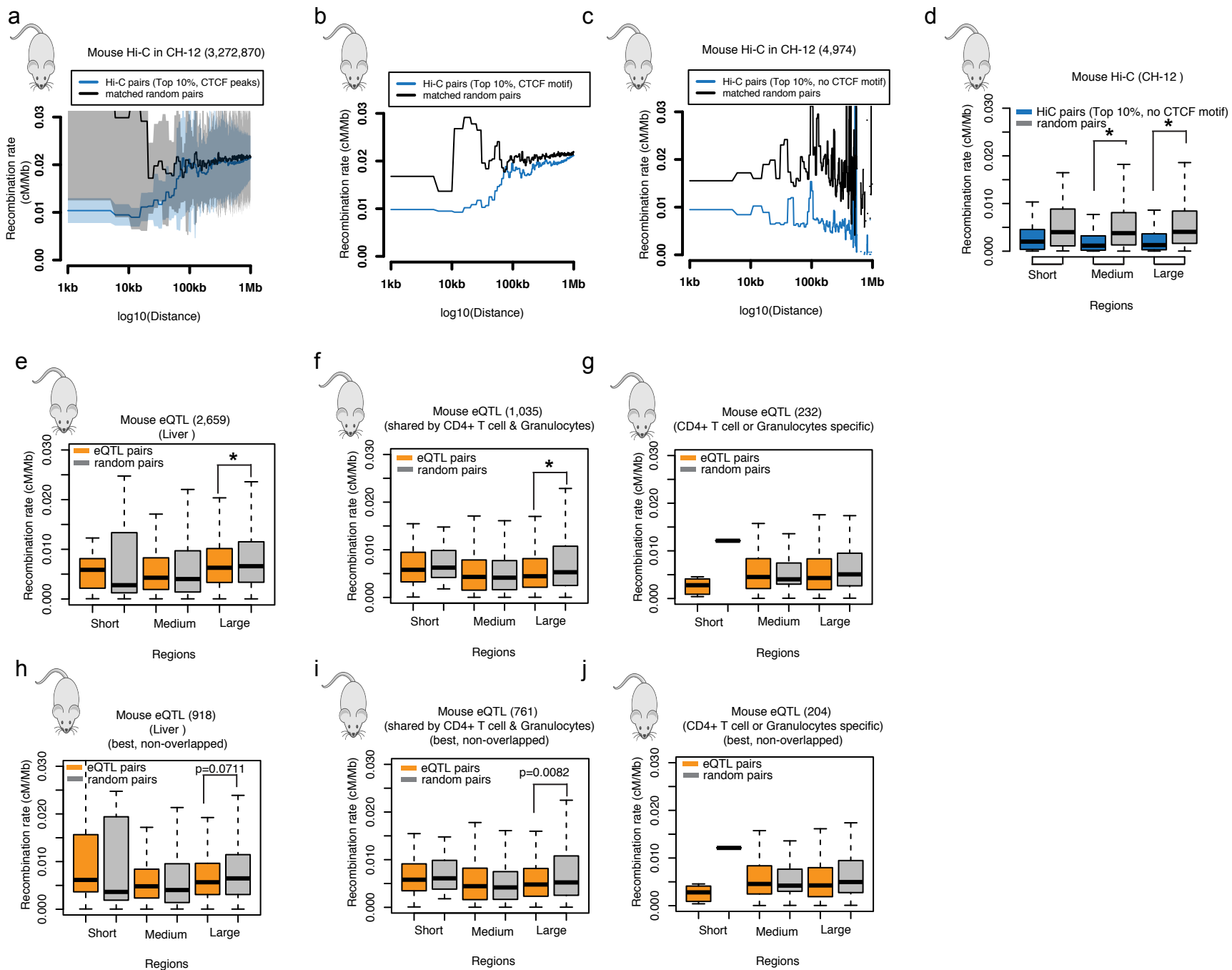

Supplement: Supplementary file 1 — Scatter plot of recombination rate within genetic, physical, and activity links. Figure S2. Recombination valleys within non-overlapped genetic, physical, and activity links. Figure S3. Differences in recombination rate between best meQTL pairs and locally adjacent pairs. Figure S4. Recombination valleys in eQTLs in different tissues and cell lines. Figure S5. Recombination valleys within functional links at different thresholds. Figure S6. Recombination valleys in different recombination rate maps. Figure S7. Recombination valleys after controlling for physical length, G + C percentage, CpG density, SNP density, PRDM9 motif frequency, gene density, and distance to TSS. Figure S8. Recombination valleys exist in intergenic regions and non-coding bases. Figure S9. Recombination rate between Hi-C pairs and matched random intervals within the same HiCCUPS loops. Figure S10. eQTL evidence supported by chromatin conformation signals in the same cell line shows stronger depletion of recombination rate. Figure S11. Relationship between recombination valleys and CTCF. Figure S12 Recombination valleys between physical links, activity links without CTCF motifs, and matched random intervals also without CTCF motifs. Figure S13. Recombination valleys are most prominent at enhancer–TSS links, DNase–TSS links Hi-C links, and ChIA-PET PolII/PolII links associated with housekeeping genes. Figure S14. Recombination valleys are prominent at early embryonic developmental genes, but not at other cell type-specific genes. Figure S15. Recombination valleys are prominent at housekeeping genes in highly expressed and minimally expressed genes at the oocyte stage. Figure S16. Recombination valleys are most prominent at constitutive eQTL links. Figure S17. Recombination valleys in mouse regulatory domains. Figure S18. Recombination valleys are correlated with hotspot density and DNA methylation. Figure S19. Mechanistic model for recombination valley in regulatory domains. Figure S20. Relation [file 13059_2017_1308_MOESM1_ESM.zip › Supplemental_Fig_S17.pdf]

# Supplementary Figure 18

a

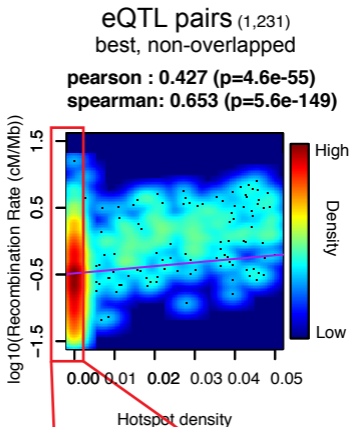

b

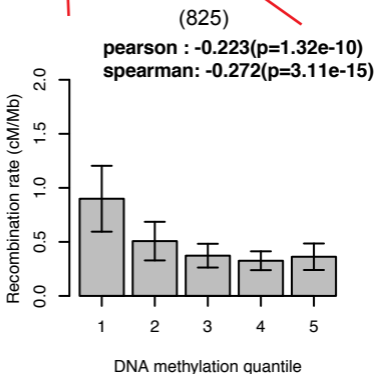

Supplement: Supplementary file 1 — Scatter plot of recombination rate within genetic, physical, and activity links. Figure S2. Recombination valleys within non-overlapped genetic, physical, and activity links. Figure S3. Differences in recombination rate between best meQTL pairs and locally adjacent pairs. Figure S4. Recombination valleys in eQTLs in different tissues and cell lines. Figure S5. Recombination valleys within functional links at different thresholds. Figure S6. Recombination valleys in different recombination rate maps. Figure S7. Recombination valleys after controlling for physical length, G + C percentage, CpG density, SNP density, PRDM9 motif frequency, gene density, and distance to TSS. Figure S8. Recombination valleys exist in intergenic regions and non-coding bases. Figure S9. Recombination rate between Hi-C pairs and matched random intervals within the same HiCCUPS loops. Figure S10. eQTL evidence supported by chromatin conformation signals in the same cell line shows stronger depletion of recombination rate. Figure S11. Relationship between recombination valleys and CTCF. Figure S12 Recombination valleys between physical links, activity links without CTCF motifs, and matched random intervals also without CTCF motifs. Figure S13. Recombination valleys are most prominent at enhancer–TSS links, DNase–TSS links Hi-C links, and ChIA-PET PolII/PolII links associated with housekeeping genes. Figure S14. Recombination valleys are prominent at early embryonic developmental genes, but not at other cell type-specific genes. Figure S15. Recombination valleys are prominent at housekeeping genes in highly expressed and minimally expressed genes at the oocyte stage. Figure S16. Recombination valleys are most prominent at constitutive eQTL links. Figure S17. Recombination valleys in mouse regulatory domains. Figure S18. Recombination valleys are correlated with hotspot density and DNA methylation. Figure S19. Mechanistic model for recombination valley in regulatory domains. Figure S20. Relation [file 13059_2017_1308_MOESM1_ESM.zip › Supplemental_Fig_S18.pdf]

Supplementary Figure 19

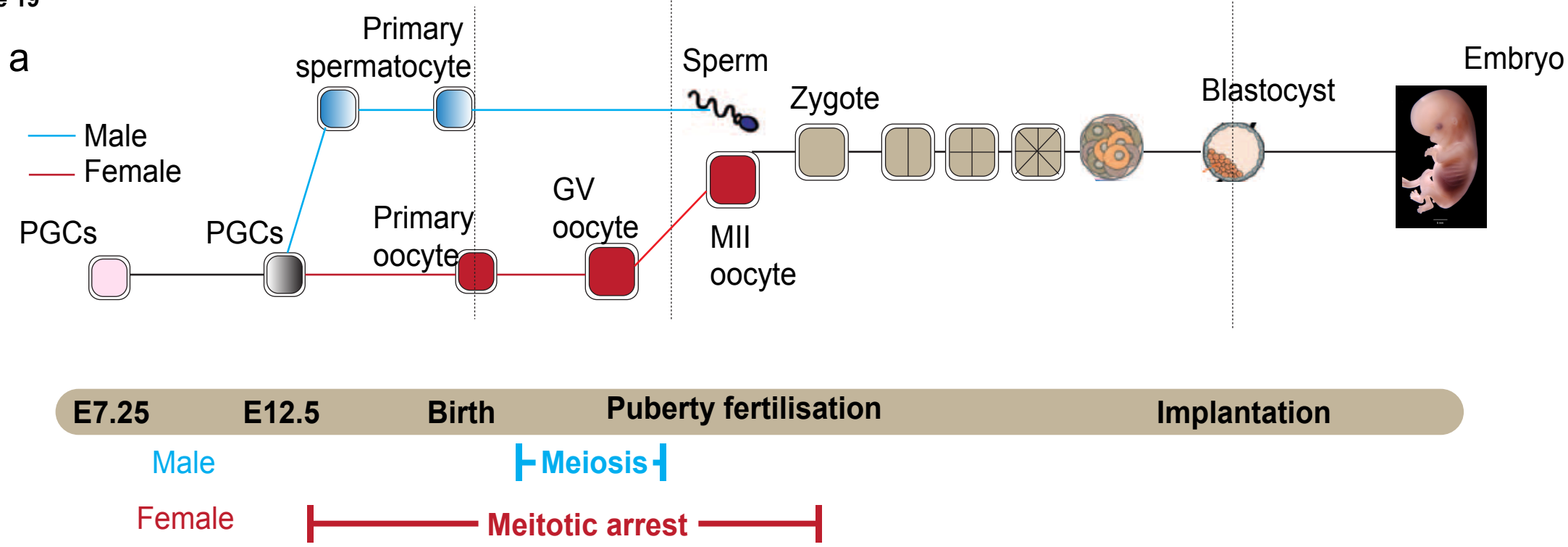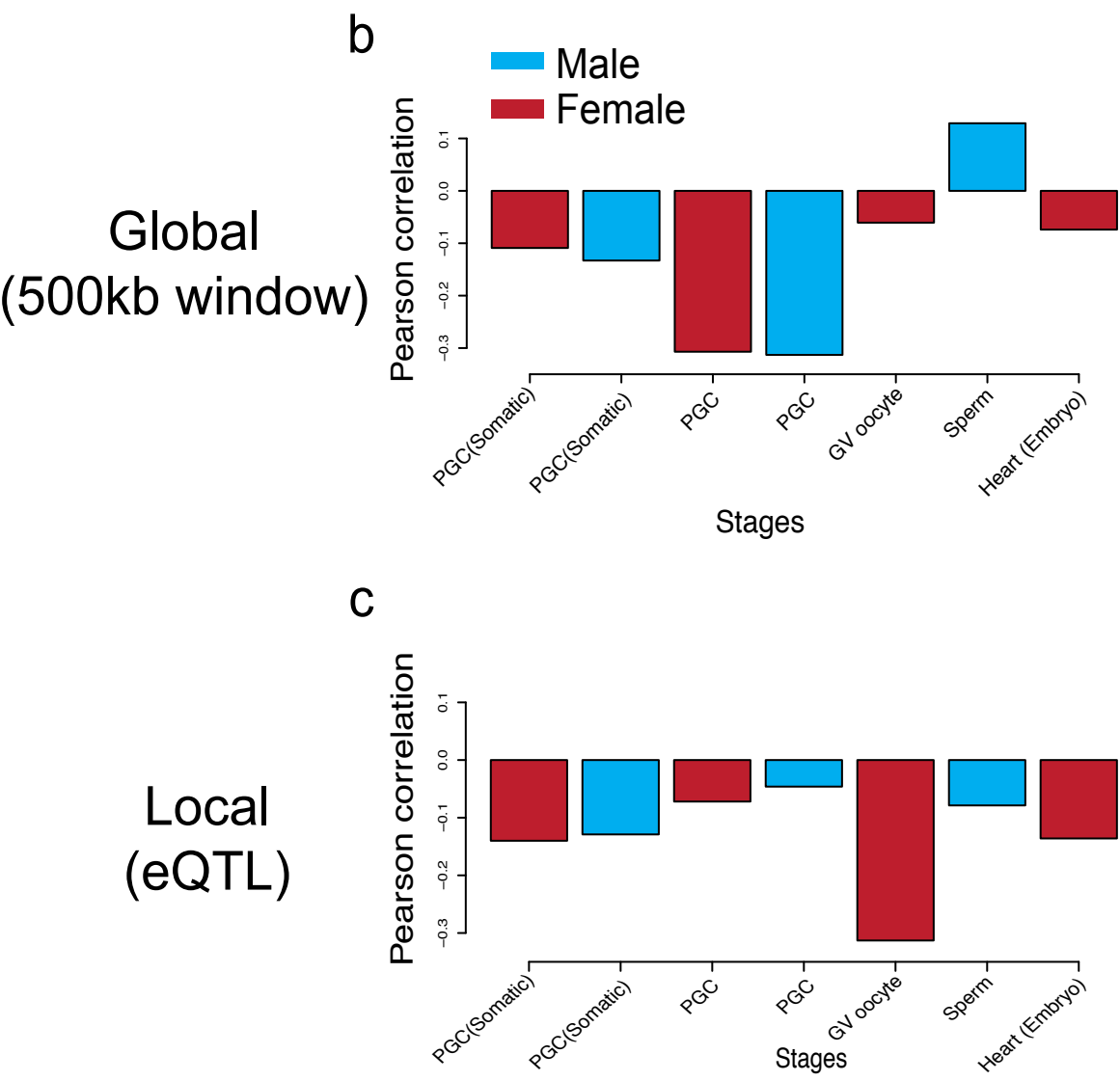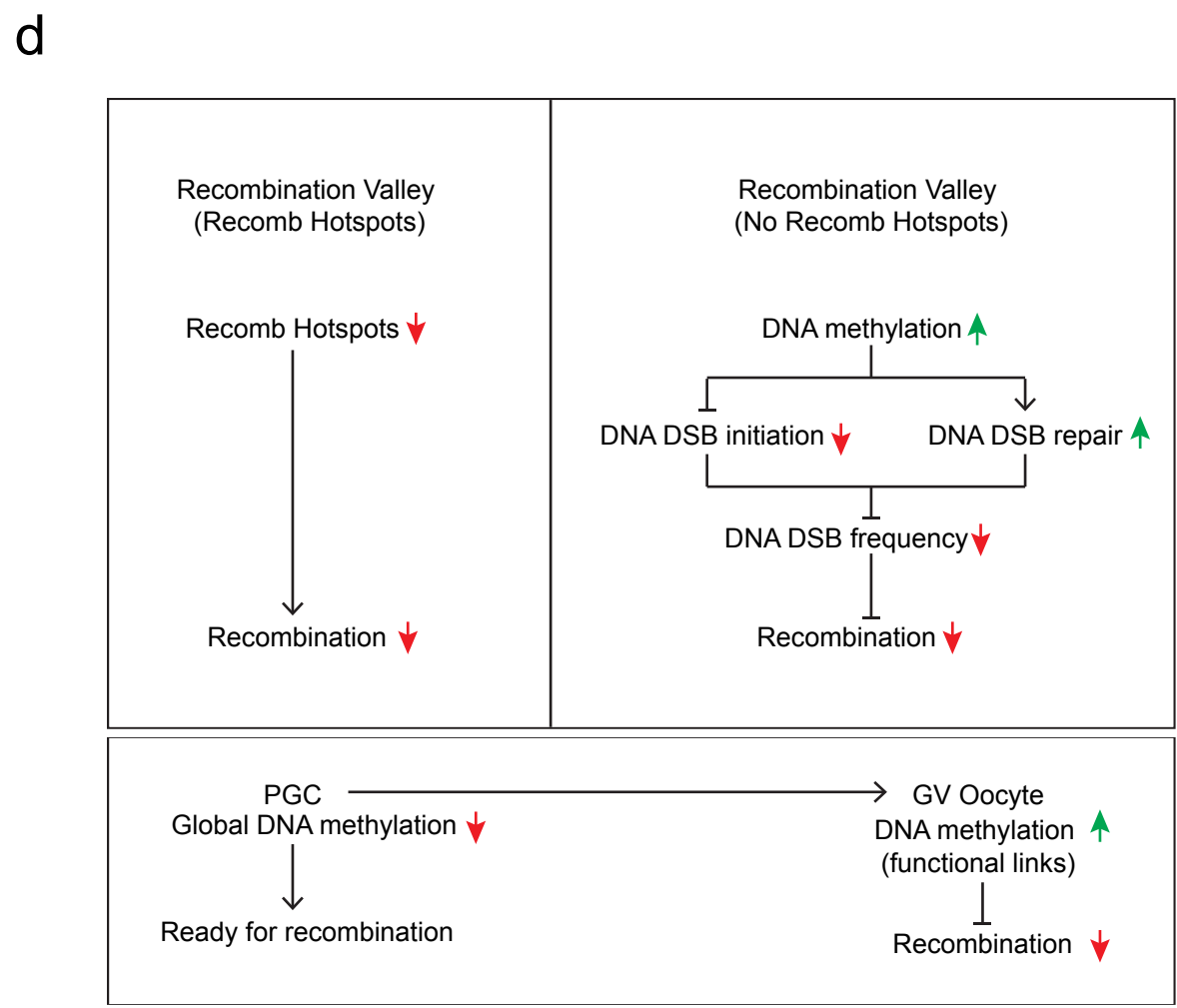

Supplement: Supplementary file 1 — Scatter plot of recombination rate within genetic, physical, and activity links. Figure S2. Recombination valleys within non-overlapped genetic, physical, and activity links. Figure S3. Differences in recombination rate between best meQTL pairs and locally adjacent pairs. Figure S4. Recombination valleys in eQTLs in different tissues and cell lines. Figure S5. Recombination valleys within functional links at different thresholds. Figure S6. Recombination valleys in different recombination rate maps. Figure S7. Recombination valleys after controlling for physical length, G + C percentage, CpG density, SNP density, PRDM9 motif frequency, gene density, and distance to TSS. Figure S8. Recombination valleys exist in intergenic regions and non-coding bases. Figure S9. Recombination rate between Hi-C pairs and matched random intervals within the same HiCCUPS loops. Figure S10. eQTL evidence supported by chromatin conformation signals in the same cell line shows stronger depletion of recombination rate. Figure S11. Relationship between recombination valleys and CTCF. Figure S12 Recombination valleys between physical links, activity links without CTCF motifs, and matched random intervals also without CTCF motifs. Figure S13. Recombination valleys are most prominent at enhancer–TSS links, DNase–TSS links Hi-C links, and ChIA-PET PolII/PolII links associated with housekeeping genes. Figure S14. Recombination valleys are prominent at early embryonic developmental genes, but not at other cell type-specific genes. Figure S15. Recombination valleys are prominent at housekeeping genes in highly expressed and minimally expressed genes at the oocyte stage. Figure S16. Recombination valleys are most prominent at constitutive eQTL links. Figure S17. Recombination valleys in mouse regulatory domains. Figure S18. Recombination valleys are correlated with hotspot density and DNA methylation. Figure S19. Mechanistic model for recombination valley in regulatory domains. Figure S20. Relation [file 13059_2017_1308_MOESM1_ESM.zip › Supplemental_Fig_S19.pdf]

# Supplementary Figure 2

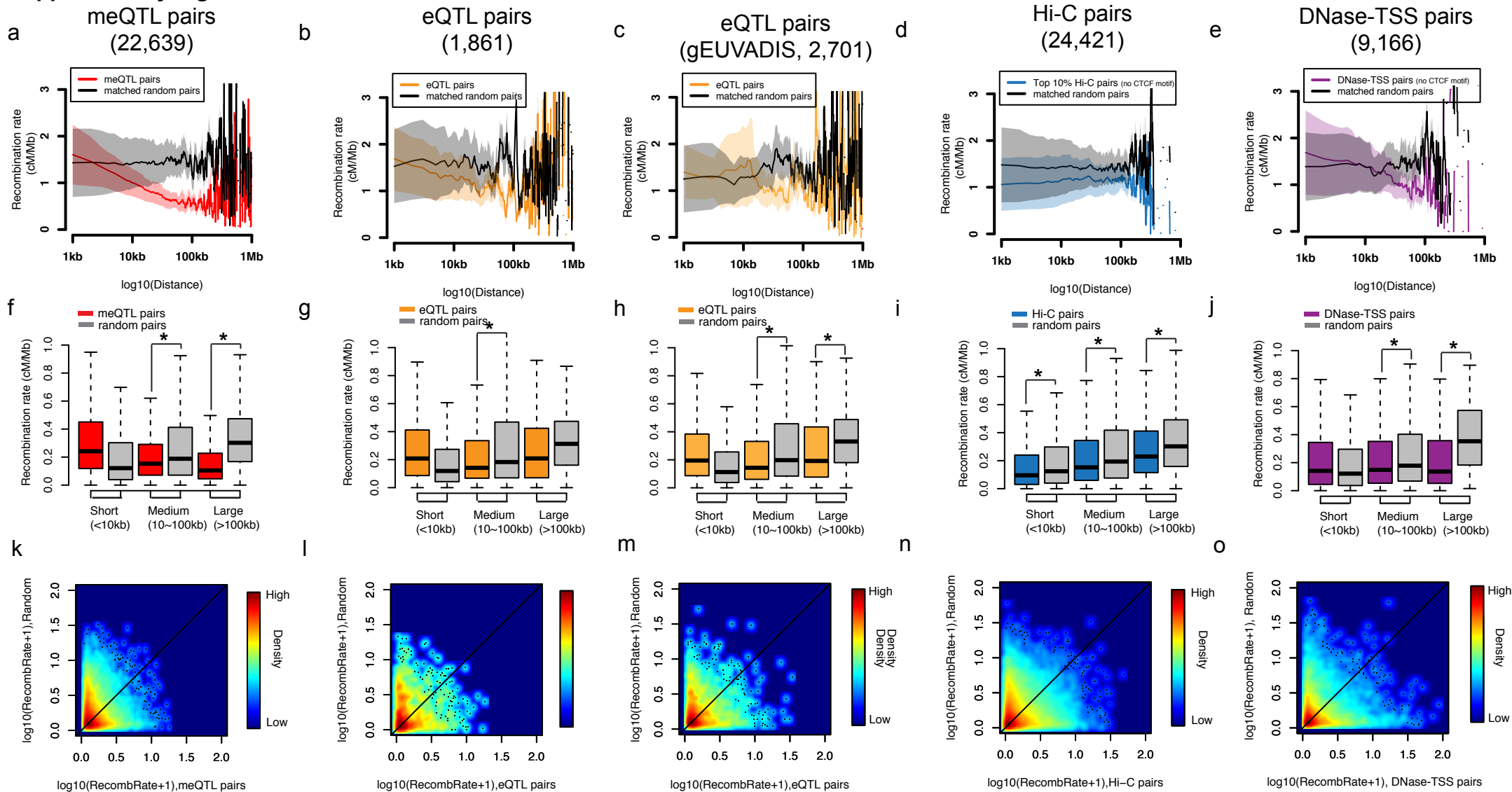

Supplement: Supplementary file 1 — Scatter plot of recombination rate within genetic, physical, and activity links. Figure S2. Recombination valleys within non-overlapped genetic, physical, and activity links. Figure S3. Differences in recombination rate between best meQTL pairs and locally adjacent pairs. Figure S4. Recombination valleys in eQTLs in different tissues and cell lines. Figure S5. Recombination valleys within functional links at different thresholds. Figure S6. Recombination valleys in different recombination rate maps. Figure S7. Recombination valleys after controlling for physical length, G + C percentage, CpG density, SNP density, PRDM9 motif frequency, gene density, and distance to TSS. Figure S8. Recombination valleys exist in intergenic regions and non-coding bases. Figure S9. Recombination rate between Hi-C pairs and matched random intervals within the same HiCCUPS loops. Figure S10. eQTL evidence supported by chromatin conformation signals in the same cell line shows stronger depletion of recombination rate. Figure S11. Relationship between recombination valleys and CTCF. Figure S12 Recombination valleys between physical links, activity links without CTCF motifs, and matched random intervals also without CTCF motifs. Figure S13. Recombination valleys are most prominent at enhancer–TSS links, DNase–TSS links Hi-C links, and ChIA-PET PolII/PolII links associated with housekeeping genes. Figure S14. Recombination valleys are prominent at early embryonic developmental genes, but not at other cell type-specific genes. Figure S15. Recombination valleys are prominent at housekeeping genes in highly expressed and minimally expressed genes at the oocyte stage. Figure S16. Recombination valleys are most prominent at constitutive eQTL links. Figure S17. Recombination valleys in mouse regulatory domains. Figure S18. Recombination valleys are correlated with hotspot density and DNA methylation. Figure S19. Mechanistic model for recombination valley in regulatory domains. Figure S20. Relation [file 13059_2017_1308_MOESM1_ESM.zip › Supplemental_Fig_S2.pdf]

Supplementary Figure 20

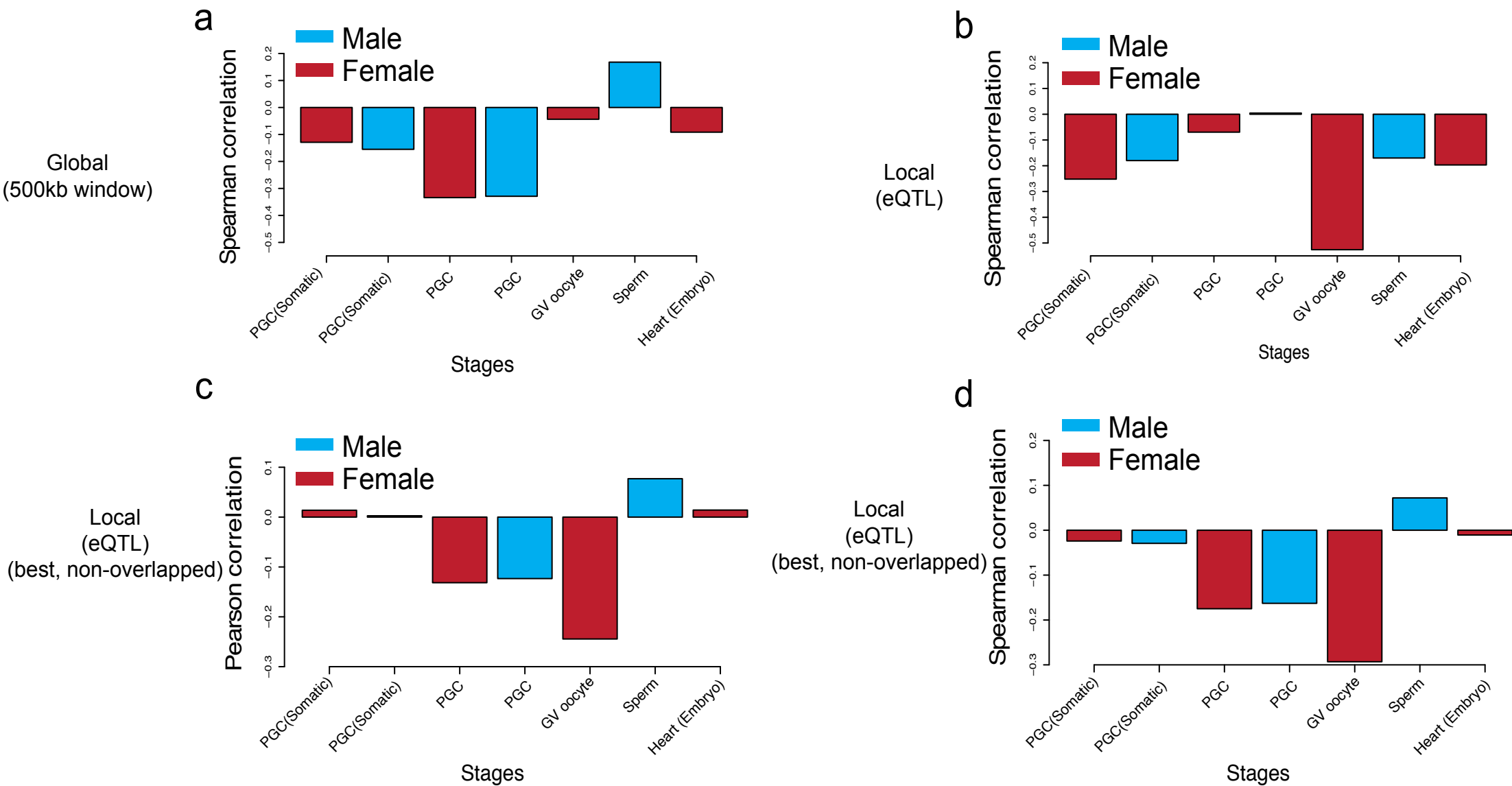

Supplement: Supplementary file 1 — Scatter plot of recombination rate within genetic, physical, and activity links. Figure S2. Recombination valleys within non-overlapped genetic, physical, and activity links. Figure S3. Differences in recombination rate between best meQTL pairs and locally adjacent pairs. Figure S4. Recombination valleys in eQTLs in different tissues and cell lines. Figure S5. Recombination valleys within functional links at different thresholds. Figure S6. Recombination valleys in different recombination rate maps. Figure S7. Recombination valleys after controlling for physical length, G + C percentage, CpG density, SNP density, PRDM9 motif frequency, gene density, and distance to TSS. Figure S8. Recombination valleys exist in intergenic regions and non-coding bases. Figure S9. Recombination rate between Hi-C pairs and matched random intervals within the same HiCCUPS loops. Figure S10. eQTL evidence supported by chromatin conformation signals in the same cell line shows stronger depletion of recombination rate. Figure S11. Relationship between recombination valleys and CTCF. Figure S12 Recombination valleys between physical links, activity links without CTCF motifs, and matched random intervals also without CTCF motifs. Figure S13. Recombination valleys are most prominent at enhancer–TSS links, DNase–TSS links Hi-C links, and ChIA-PET PolII/PolII links associated with housekeeping genes. Figure S14. Recombination valleys are prominent at early embryonic developmental genes, but not at other cell type-specific genes. Figure S15. Recombination valleys are prominent at housekeeping genes in highly expressed and minimally expressed genes at the oocyte stage. Figure S16. Recombination valleys are most prominent at constitutive eQTL links. Figure S17. Recombination valleys in mouse regulatory domains. Figure S18. Recombination valleys are correlated with hotspot density and DNA methylation. Figure S19. Mechanistic model for recombination valley in regulatory domains. Figure S20. Relation [file 13059_2017_1308_MOESM1_ESM.zip › Supplemental_Fig_S20.pdf]

Supplementary Figure 22

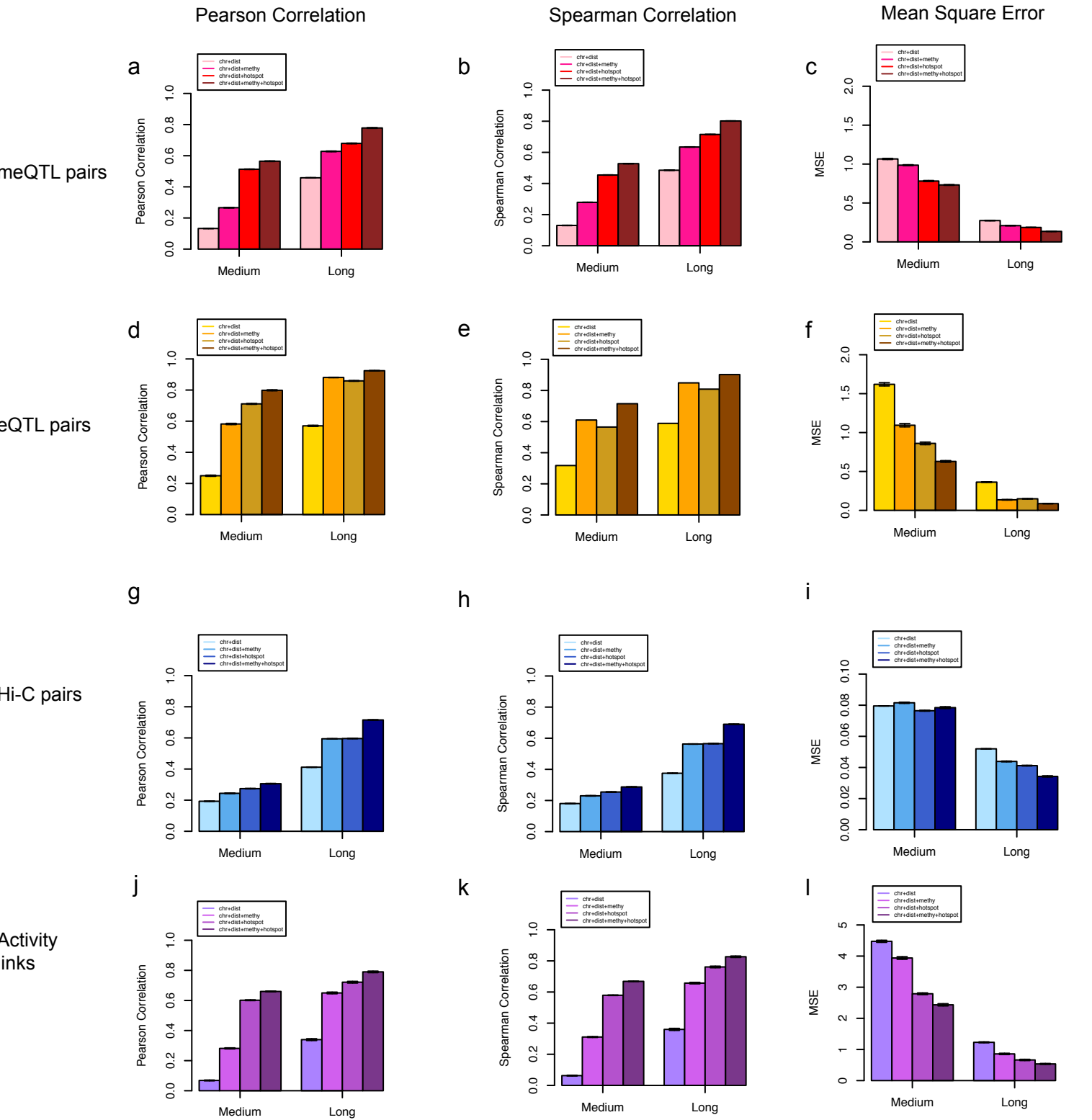

Supplement: Supplementary file 1 — Scatter plot of recombination rate within genetic, physical, and activity links. Figure S2. Recombination valleys within non-overlapped genetic, physical, and activity links. Figure S3. Differences in recombination rate between best meQTL pairs and locally adjacent pairs. Figure S4. Recombination valleys in eQTLs in different tissues and cell lines. Figure S5. Recombination valleys within functional links at different thresholds. Figure S6. Recombination valleys in different recombination rate maps. Figure S7. Recombination valleys after controlling for physical length, G + C percentage, CpG density, SNP density, PRDM9 motif frequency, gene density, and distance to TSS. Figure S8. Recombination valleys exist in intergenic regions and non-coding bases. Figure S9. Recombination rate between Hi-C pairs and matched random intervals within the same HiCCUPS loops. Figure S10. eQTL evidence supported by chromatin conformation signals in the same cell line shows stronger depletion of recombination rate. Figure S11. Relationship between recombination valleys and CTCF. Figure S12 Recombination valleys between physical links, activity links without CTCF motifs, and matched random intervals also without CTCF motifs. Figure S13. Recombination valleys are most prominent at enhancer–TSS links, DNase–TSS links Hi-C links, and ChIA-PET PolII/PolII links associated with housekeeping genes. Figure S14. Recombination valleys are prominent at early embryonic developmental genes, but not at other cell type-specific genes. Figure S15. Recombination valleys are prominent at housekeeping genes in highly expressed and minimally expressed genes at the oocyte stage. Figure S16. Recombination valleys are most prominent at constitutive eQTL links. Figure S17. Recombination valleys in mouse regulatory domains. Figure S18. Recombination valleys are correlated with hotspot density and DNA methylation. Figure S19. Mechanistic model for recombination valley in regulatory domains. Figure S20. Relation [file 13059_2017_1308_MOESM1_ESM.zip › Supplemental_Fig_S22.pdf]

# Supplementary Figure 3

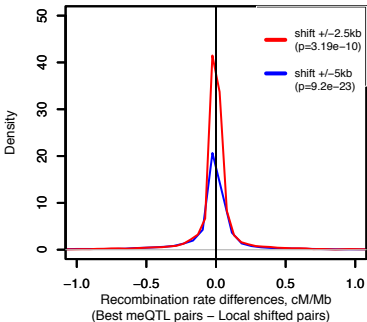

Supplement: Supplementary file 1 — Scatter plot of recombination rate within genetic, physical, and activity links. Figure S2. Recombination valleys within non-overlapped genetic, physical, and activity links. Figure S3. Differences in recombination rate between best meQTL pairs and locally adjacent pairs. Figure S4. Recombination valleys in eQTLs in different tissues and cell lines. Figure S5. Recombination valleys within functional links at different thresholds. Figure S6. Recombination valleys in different recombination rate maps. Figure S7. Recombination valleys after controlling for physical length, G + C percentage, CpG density, SNP density, PRDM9 motif frequency, gene density, and distance to TSS. Figure S8. Recombination valleys exist in intergenic regions and non-coding bases. Figure S9. Recombination rate between Hi-C pairs and matched random intervals within the same HiCCUPS loops. Figure S10. eQTL evidence supported by chromatin conformation signals in the same cell line shows stronger depletion of recombination rate. Figure S11. Relationship between recombination valleys and CTCF. Figure S12 Recombination valleys between physical links, activity links without CTCF motifs, and matched random intervals also without CTCF motifs. Figure S13. Recombination valleys are most prominent at enhancer–TSS links, DNase–TSS links Hi-C links, and ChIA-PET PolII/PolII links associated with housekeeping genes. Figure S14. Recombination valleys are prominent at early embryonic developmental genes, but not at other cell type-specific genes. Figure S15. Recombination valleys are prominent at housekeeping genes in highly expressed and minimally expressed genes at the oocyte stage. Figure S16. Recombination valleys are most prominent at constitutive eQTL links. Figure S17. Recombination valleys in mouse regulatory domains. Figure S18. Recombination valleys are correlated with hotspot density and DNA methylation. Figure S19. Mechanistic model for recombination valley in regulatory domains. Figure S20. Relation [file 13059_2017_1308_MOESM1_ESM.zip › Supplemental_Fig_S3.pdf]

# Supplementary Figure 4

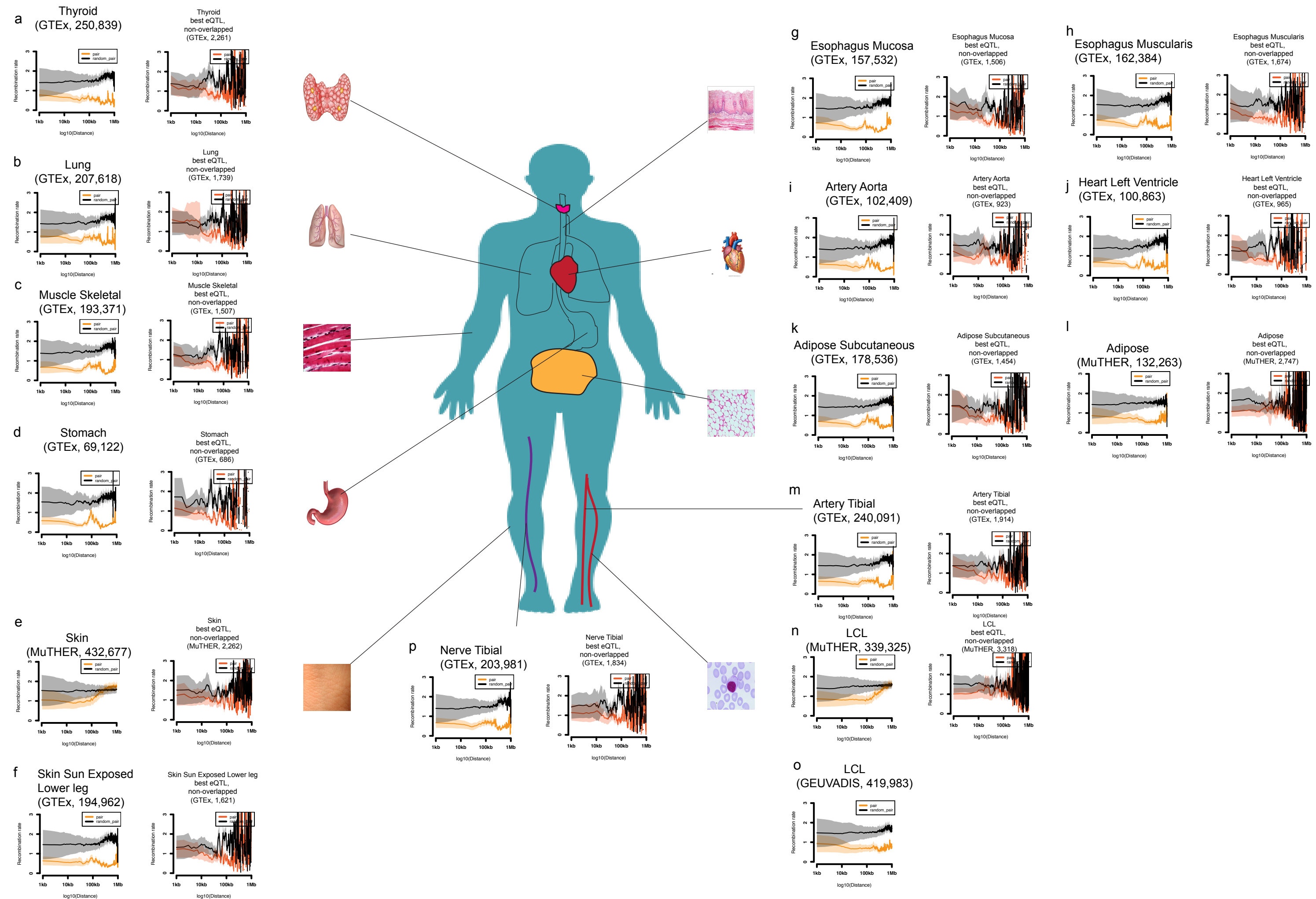

Supplement: Supplementary file 1 — Scatter plot of recombination rate within genetic, physical, and activity links. Figure S2. Recombination valleys within non-overlapped genetic, physical, and activity links. Figure S3. Differences in recombination rate between best meQTL pairs and locally adjacent pairs. Figure S4. Recombination valleys in eQTLs in different tissues and cell lines. Figure S5. Recombination valleys within functional links at different thresholds. Figure S6. Recombination valleys in different recombination rate maps. Figure S7. Recombination valleys after controlling for physical length, G + C percentage, CpG density, SNP density, PRDM9 motif frequency, gene density, and distance to TSS. Figure S8. Recombination valleys exist in intergenic regions and non-coding bases. Figure S9. Recombination rate between Hi-C pairs and matched random intervals within the same HiCCUPS loops. Figure S10. eQTL evidence supported by chromatin conformation signals in the same cell line shows stronger depletion of recombination rate. Figure S11. Relationship between recombination valleys and CTCF. Figure S12 Recombination valleys between physical links, activity links without CTCF motifs, and matched random intervals also without CTCF motifs. Figure S13. Recombination valleys are most prominent at enhancer–TSS links, DNase–TSS links Hi-C links, and ChIA-PET PolII/PolII links associated with housekeeping genes. Figure S14. Recombination valleys are prominent at early embryonic developmental genes, but not at other cell type-specific genes. Figure S15. Recombination valleys are prominent at housekeeping genes in highly expressed and minimally expressed genes at the oocyte stage. Figure S16. Recombination valleys are most prominent at constitutive eQTL links. Figure S17. Recombination valleys in mouse regulatory domains. Figure S18. Recombination valleys are correlated with hotspot density and DNA methylation. Figure S19. Mechanistic model for recombination valley in regulatory domains. Figure S20. Relation [file 13059_2017_1308_MOESM1_ESM.zip › Supplemental_Fig_S4.pdf]

# Supplementary Figure 5

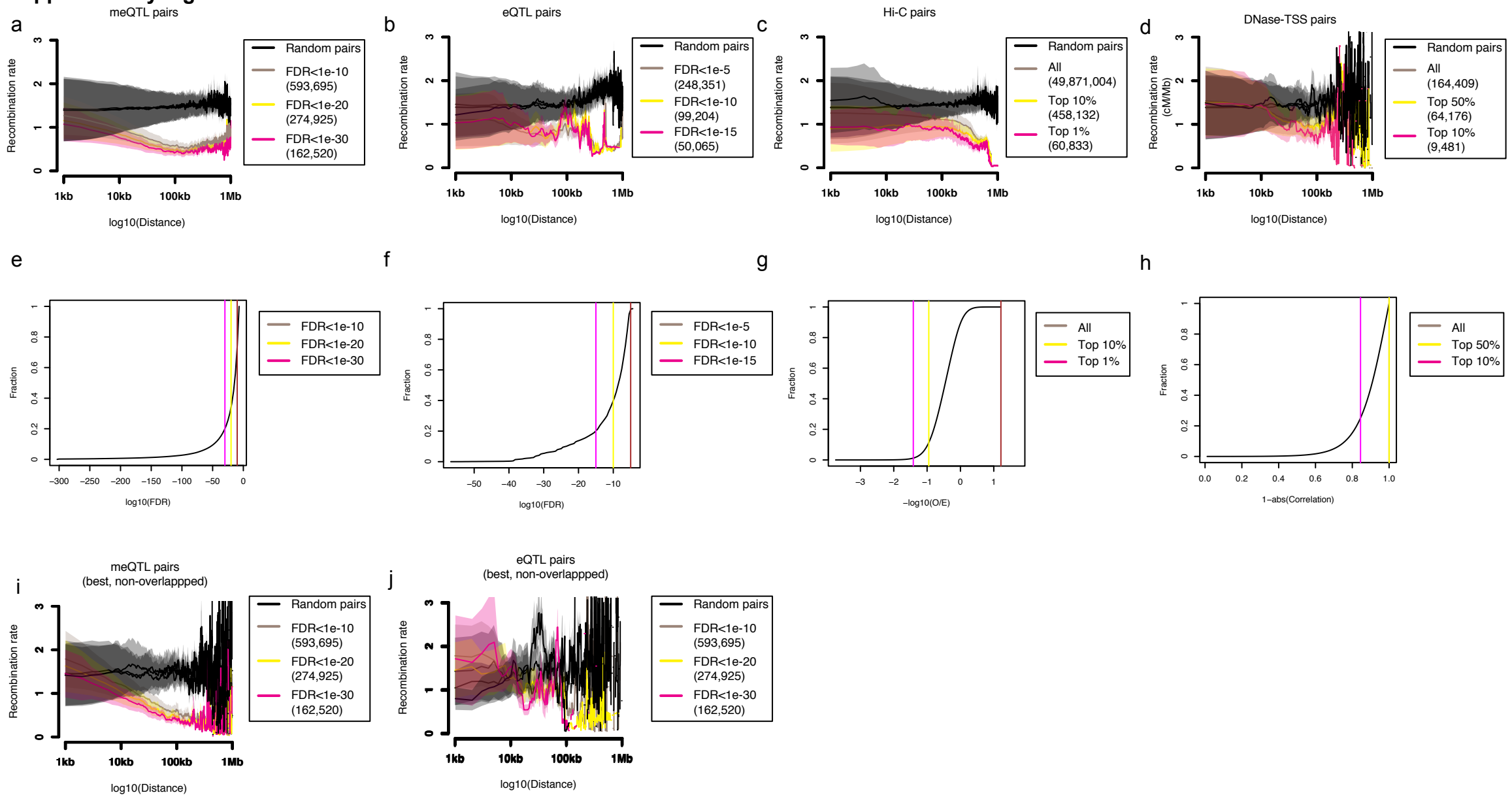

Supplement: Supplementary file 1 — Scatter plot of recombination rate within genetic, physical, and activity links. Figure S2. Recombination valleys within non-overlapped genetic, physical, and activity links. Figure S3. Differences in recombination rate between best meQTL pairs and locally adjacent pairs. Figure S4. Recombination valleys in eQTLs in different tissues and cell lines. Figure S5. Recombination valleys within functional links at different thresholds. Figure S6. Recombination valleys in different recombination rate maps. Figure S7. Recombination valleys after controlling for physical length, G + C percentage, CpG density, SNP density, PRDM9 motif frequency, gene density, and distance to TSS. Figure S8. Recombination valleys exist in intergenic regions and non-coding bases. Figure S9. Recombination rate between Hi-C pairs and matched random intervals within the same HiCCUPS loops. Figure S10. eQTL evidence supported by chromatin conformation signals in the same cell line shows stronger depletion of recombination rate. Figure S11. Relationship between recombination valleys and CTCF. Figure S12 Recombination valleys between physical links, activity links without CTCF motifs, and matched random intervals also without CTCF motifs. Figure S13. Recombination valleys are most prominent at enhancer–TSS links, DNase–TSS links Hi-C links, and ChIA-PET PolII/PolII links associated with housekeeping genes. Figure S14. Recombination valleys are prominent at early embryonic developmental genes, but not at other cell type-specific genes. Figure S15. Recombination valleys are prominent at housekeeping genes in highly expressed and minimally expressed genes at the oocyte stage. Figure S16. Recombination valleys are most prominent at constitutive eQTL links. Figure S17. Recombination valleys in mouse regulatory domains. Figure S18. Recombination valleys are correlated with hotspot density and DNA methylation. Figure S19. Mechanistic model for recombination valley in regulatory domains. Figure S20. Relation [file 13059_2017_1308_MOESM1_ESM.zip › Supplemental_Fig_S5.pdf]

**Supplementary Figure 6**

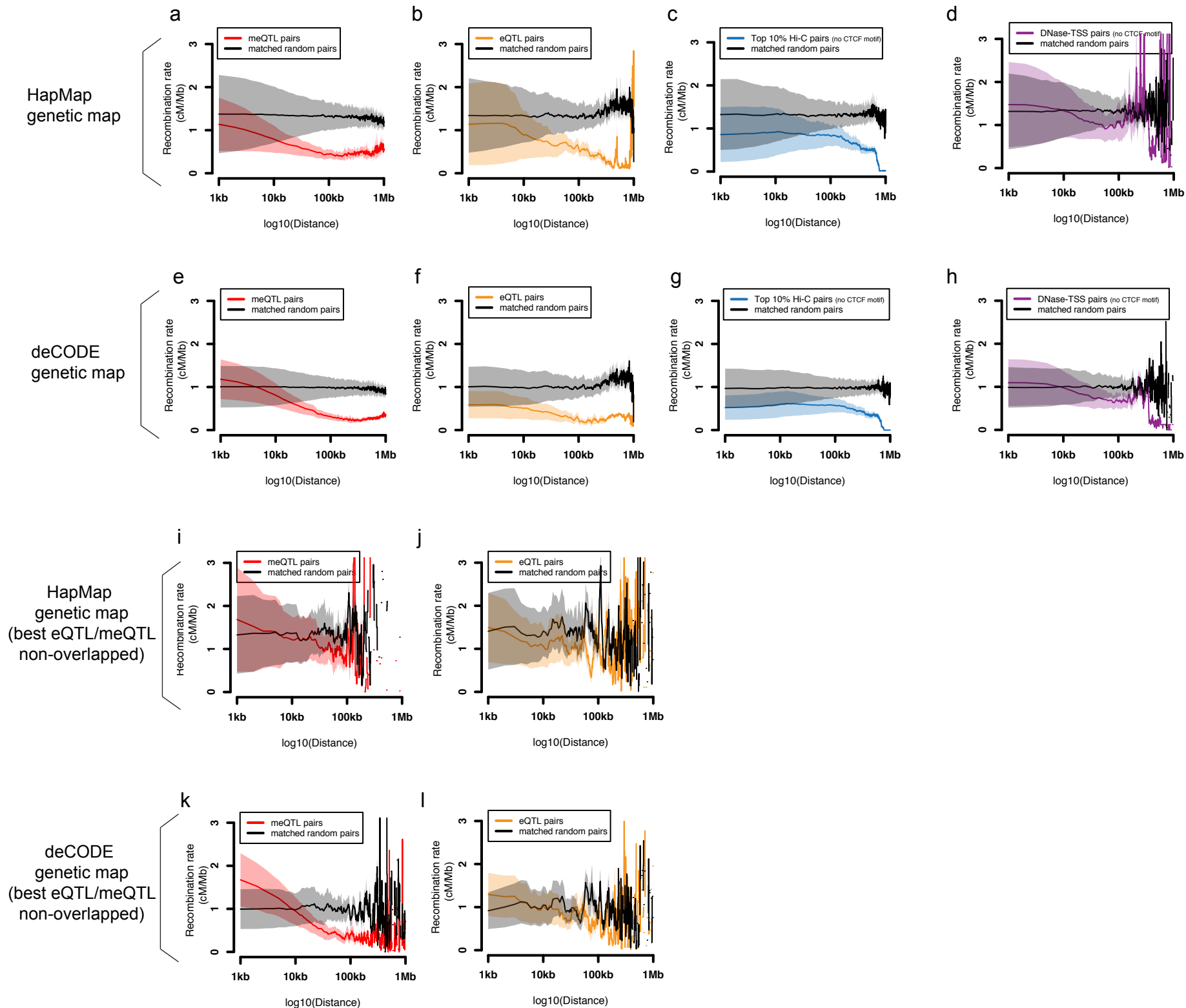

Supplement: Supplementary file 1 — Scatter plot of recombination rate within genetic, physical, and activity links. Figure S2. Recombination valleys within non-overlapped genetic, physical, and activity links. Figure S3. Differences in recombination rate between best meQTL pairs and locally adjacent pairs. Figure S4. Recombination valleys in eQTLs in different tissues and cell lines. Figure S5. Recombination valleys within functional links at different thresholds. Figure S6. Recombination valleys in different recombination rate maps. Figure S7. Recombination valleys after controlling for physical length, G + C percentage, CpG density, SNP density, PRDM9 motif frequency, gene density, and distance to TSS. Figure S8. Recombination valleys exist in intergenic regions and non-coding bases. Figure S9. Recombination rate between Hi-C pairs and matched random intervals within the same HiCCUPS loops. Figure S10. eQTL evidence supported by chromatin conformation signals in the same cell line shows stronger depletion of recombination rate. Figure S11. Relationship between recombination valleys and CTCF. Figure S12 Recombination valleys between physical links, activity links without CTCF motifs, and matched random intervals also without CTCF motifs. Figure S13. Recombination valleys are most prominent at enhancer–TSS links, DNase–TSS links Hi-C links, and ChIA-PET PolII/PolII links associated with housekeeping genes. Figure S14. Recombination valleys are prominent at early embryonic developmental genes, but not at other cell type-specific genes. Figure S15. Recombination valleys are prominent at housekeeping genes in highly expressed and minimally expressed genes at the oocyte stage. Figure S16. Recombination valleys are most prominent at constitutive eQTL links. Figure S17. Recombination valleys in mouse regulatory domains. Figure S18. Recombination valleys are correlated with hotspot density and DNA methylation. Figure S19. Mechanistic model for recombination valley in regulatory domains. Figure S20. Relation [file 13059_2017_1308_MOESM1_ESM.zip › Supplemental_Fig_S6.pdf]

**Supplementary Figure 7**

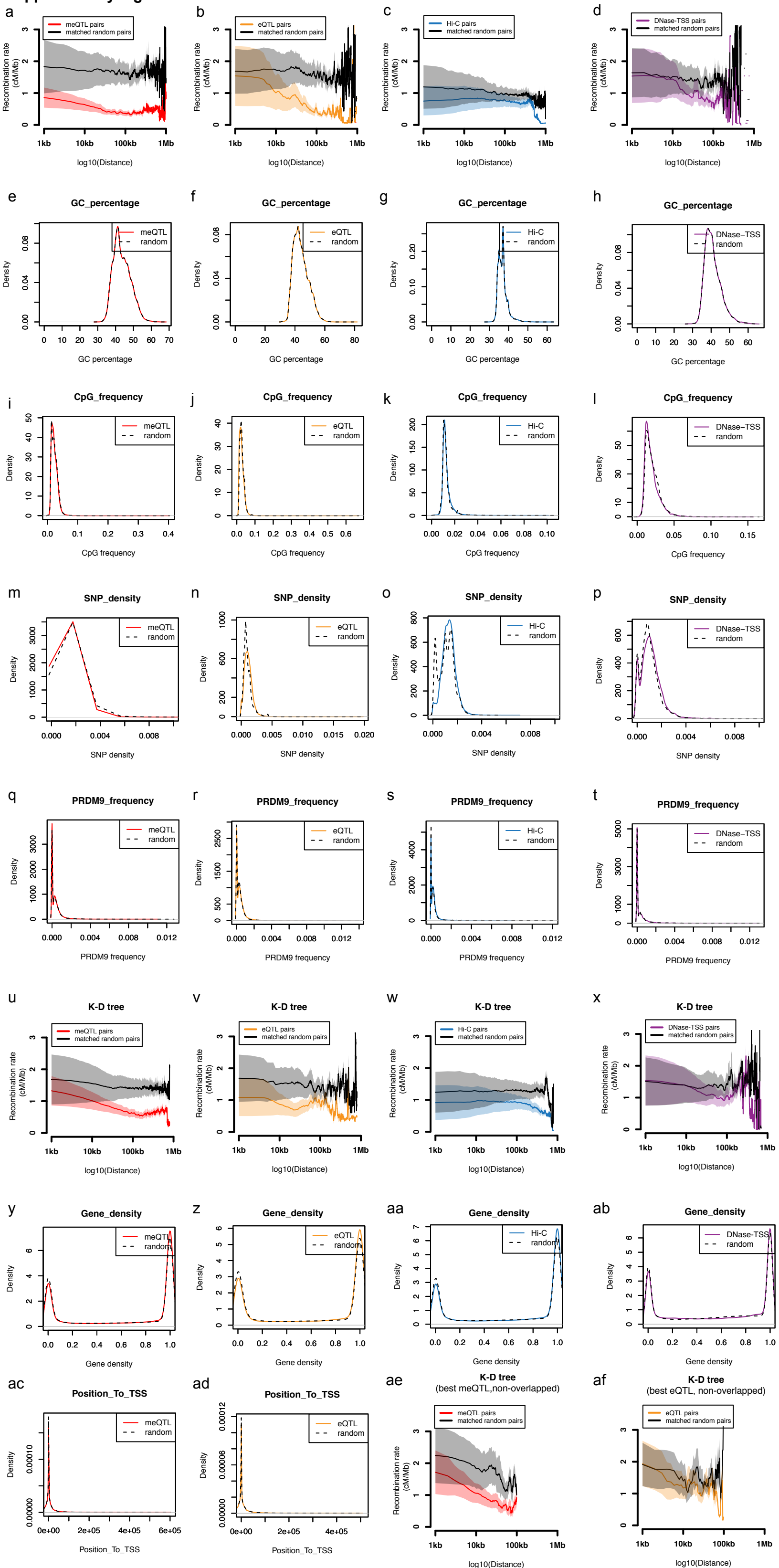

Supplement: Supplementary file 1 — Scatter plot of recombination rate within genetic, physical, and activity links. Figure S2. Recombination valleys within non-overlapped genetic, physical, and activity links. Figure S3. Differences in recombination rate between best meQTL pairs and locally adjacent pairs. Figure S4. Recombination valleys in eQTLs in different tissues and cell lines. Figure S5. Recombination valleys within functional links at different thresholds. Figure S6. Recombination valleys in different recombination rate maps. Figure S7. Recombination valleys after controlling for physical length, G + C percentage, CpG density, SNP density, PRDM9 motif frequency, gene density, and distance to TSS. Figure S8. Recombination valleys exist in intergenic regions and non-coding bases. Figure S9. Recombination rate between Hi-C pairs and matched random intervals within the same HiCCUPS loops. Figure S10. eQTL evidence supported by chromatin conformation signals in the same cell line shows stronger depletion of recombination rate. Figure S11. Relationship between recombination valleys and CTCF. Figure S12 Recombination valleys between physical links, activity links without CTCF motifs, and matched random intervals also without CTCF motifs. Figure S13. Recombination valleys are most prominent at enhancer–TSS links, DNase–TSS links Hi-C links, and ChIA-PET PolII/PolII links associated with housekeeping genes. Figure S14. Recombination valleys are prominent at early embryonic developmental genes, but not at other cell type-specific genes. Figure S15. Recombination valleys are prominent at housekeeping genes in highly expressed and minimally expressed genes at the oocyte stage. Figure S16. Recombination valleys are most prominent at constitutive eQTL links. Figure S17. Recombination valleys in mouse regulatory domains. Figure S18. Recombination valleys are correlated with hotspot density and DNA methylation. Figure S19. Mechanistic model for recombination valley in regulatory domains. Figure S20. Relation [file 13059_2017_1308_MOESM1_ESM.zip › Supplemental_Fig_S7.pdf]

# Supplementary Figure 8

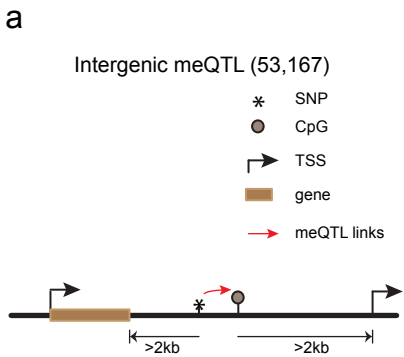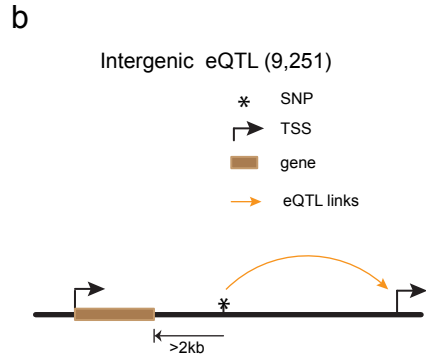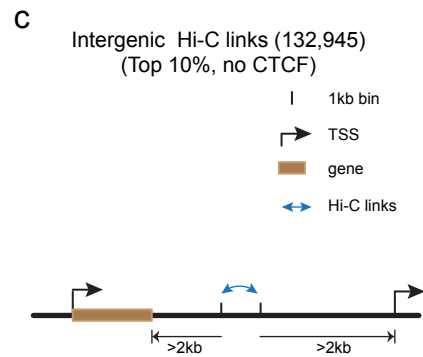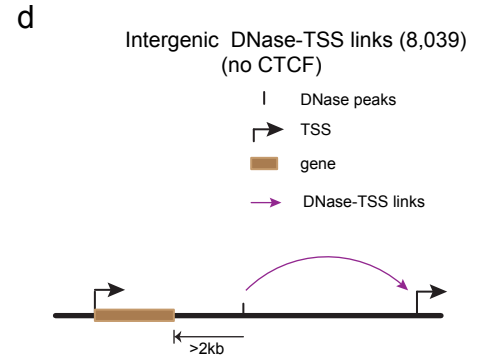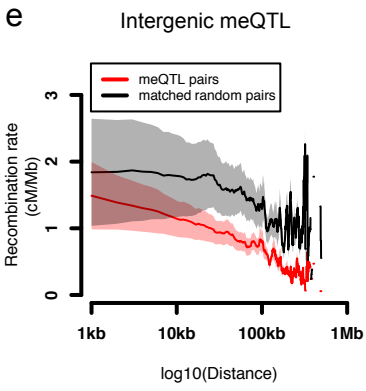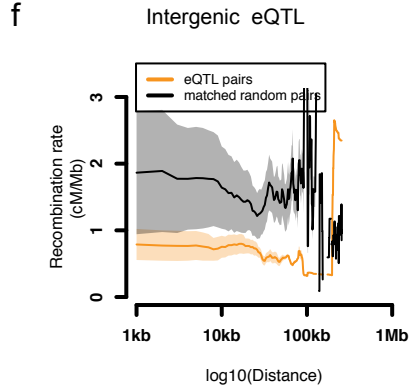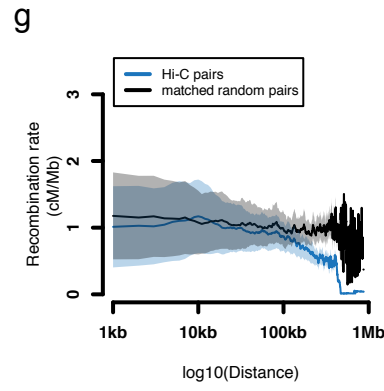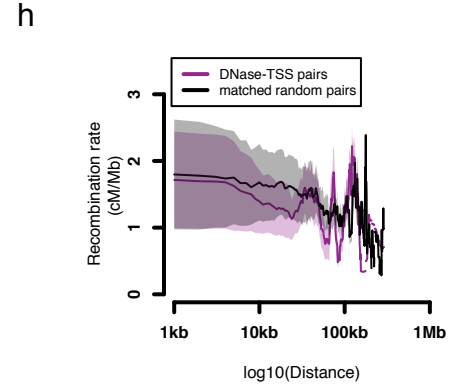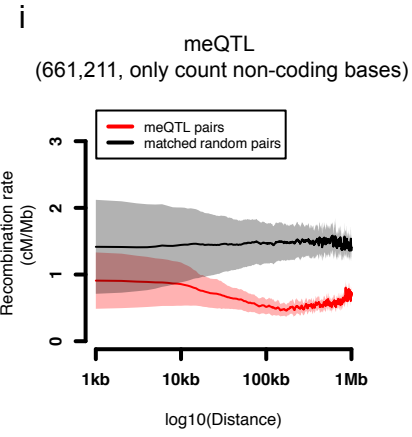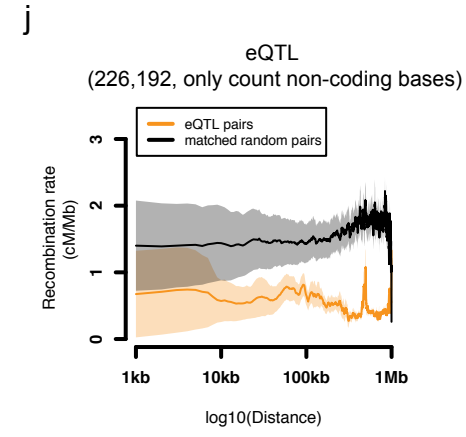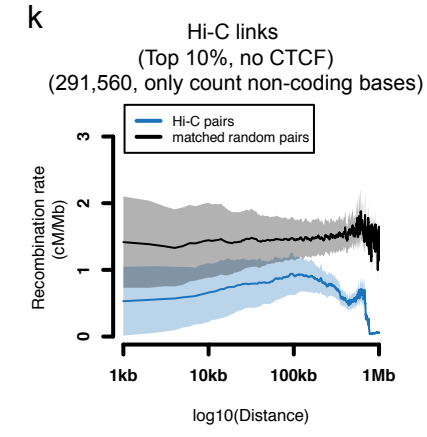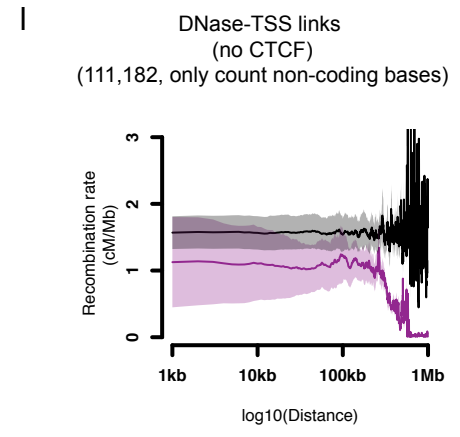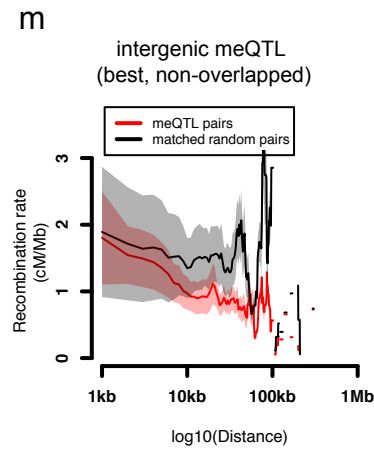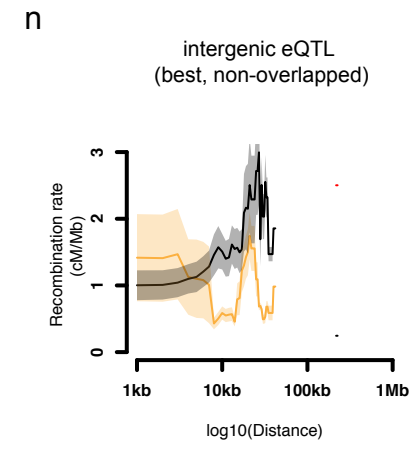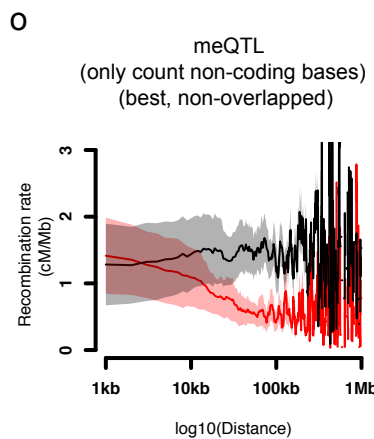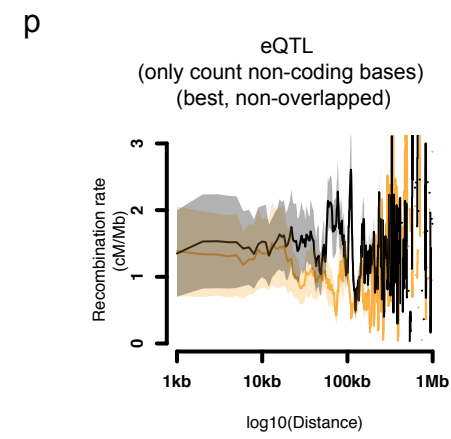

Supplement: Supplementary file 1 — Scatter plot of recombination rate within genetic, physical, and activity links. Figure S2. Recombination valleys within non-overlapped genetic, physical, and activity links. Figure S3. Differences in recombination rate between best meQTL pairs and locally adjacent pairs. Figure S4. Recombination valleys in eQTLs in different tissues and cell lines. Figure S5. Recombination valleys within functional links at different thresholds. Figure S6. Recombination valleys in different recombination rate maps. Figure S7. Recombination valleys after controlling for physical length, G + C percentage, CpG density, SNP density, PRDM9 motif frequency, gene density, and distance to TSS. Figure S8. Recombination valleys exist in intergenic regions and non-coding bases. Figure S9. Recombination rate between Hi-C pairs and matched random intervals within the same HiCCUPS loops. Figure S10. eQTL evidence supported by chromatin conformation signals in the same cell line shows stronger depletion of recombination rate. Figure S11. Relationship between recombination valleys and CTCF. Figure S12 Recombination valleys between physical links, activity links without CTCF motifs, and matched random intervals also without CTCF motifs. Figure S13. Recombination valleys are most prominent at enhancer–TSS links, DNase–TSS links Hi-C links, and ChIA-PET PolII/PolII links associated with housekeeping genes. Figure S14. Recombination valleys are prominent at early embryonic developmental genes, but not at other cell type-specific genes. Figure S15. Recombination valleys are prominent at housekeeping genes in highly expressed and minimally expressed genes at the oocyte stage. Figure S16. Recombination valleys are most prominent at constitutive eQTL links. Figure S17. Recombination valleys in mouse regulatory domains. Figure S18. Recombination valleys are correlated with hotspot density and DNA methylation. Figure S19. Mechanistic model for recombination valley in regulatory domains. Figure S20. Relation [file 13059_2017_1308_MOESM1_ESM.zip › Supplemental_Fig_S8.pdf]

# Supplementary Figure 9

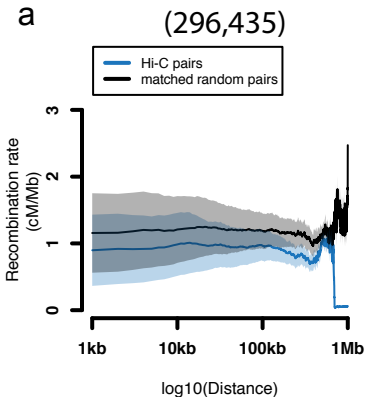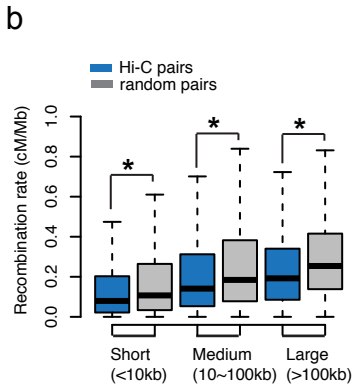

Supplement: Supplementary file 1 — Scatter plot of recombination rate within genetic, physical, and activity links. Figure S2. Recombination valleys within non-overlapped genetic, physical, and activity links. Figure S3. Differences in recombination rate between best meQTL pairs and locally adjacent pairs. Figure S4. Recombination valleys in eQTLs in different tissues and cell lines. Figure S5. Recombination valleys within functional links at different thresholds. Figure S6. Recombination valleys in different recombination rate maps. Figure S7. Recombination valleys after controlling for physical length, G + C percentage, CpG density, SNP density, PRDM9 motif frequency, gene density, and distance to TSS. Figure S8. Recombination valleys exist in intergenic regions and non-coding bases. Figure S9. Recombination rate between Hi-C pairs and matched random intervals within the same HiCCUPS loops. Figure S10. eQTL evidence supported by chromatin conformation signals in the same cell line shows stronger depletion of recombination rate. Figure S11. Relationship between recombination valleys and CTCF. Figure S12 Recombination valleys between physical links, activity links without CTCF motifs, and matched random intervals also without CTCF motifs. Figure S13. Recombination valleys are most prominent at enhancer–TSS links, DNase–TSS links Hi-C links, and ChIA-PET PolII/PolII links associated with housekeeping genes. Figure S14. Recombination valleys are prominent at early embryonic developmental genes, but not at other cell type-specific genes. Figure S15. Recombination valleys are prominent at housekeeping genes in highly expressed and minimally expressed genes at the oocyte stage. Figure S16. Recombination valleys are most prominent at constitutive eQTL links. Figure S17. Recombination valleys in mouse regulatory domains. Figure S18. Recombination valleys are correlated with hotspot density and DNA methylation. Figure S19. Mechanistic model for recombination valley in regulatory domains. Figure S20. Relation [file 13059_2017_1308_MOESM1_ESM.zip › Supplemental_Fig_S9.pdf]
