# Supplementary material for: Evidence of reduced recombination rate in human regulatory domains
Source: Genome Biol. 2017 Oct 20;18:193. doi: 10.1186/s13059-017-1308-x (PMC5651596; doi:10.1186/s13059-017-1308-x)
Supplement: Supplementary file 2 — Supplementary methods. (DOCX 65 kb) [file 13059_2017_1308_MOESM2_ESM.docx]

Evidence of a recombination rate valley in human regulatory domains

Yaping Liu^1,2^, Abhishek Sarkar^1,2^, Pouya Kheradpour^1,2^, Jason Ernst^3^, Manolis Kellis^1,2 *^

1. Computer Science and Artificial Intelligence Lab (CSAIL), Massachusetts Institute of Technology

2. Broad Institute of MIT and Harvard, Cambridge, Massachusetts

3. Department of Biological Chemistry, David Geffen School of Medicine, University of California at Los Angeles, California, USA

* Corresponding author

Correspondence should be addressed to [manoli@mit.edu](mailto:manoli@mit.edu)

**Supplementary methods**

1. **Pruning overlapped functional links**

Bedtools was used to find the overlapped links within each set of links by the command “intersectBed -loj”. For each link, only a single overlapped link was randomly selected into the final list. If there was any overlap with links in the final list, the link was omitted during the random selection. The detail is implemented in perl script “select_non_overlap.pl”.

2. **Local random shift in genetic links**

For each best meQTL link (meQTL with smallest FDR for each CpG) after overlap pruning as in Supplementary method 4, one genomic interval with exactly the same physical length was generated randomly around +/- 5kb of the original meQTL links by the perl script “select_meqtl_pairs.pl”. A two-way paired Mann-Whitney U test was used to test the significance level of recombination rate differences between genetic links and matched local random pairs. The same step was repeated in +/- 2.5kb, and the results were largely unchanged. It is difficult to define the exact boundary of eQTL’s target regions; an eQTL’s target region in each gene could be different, such as a promoter or alternative splicing sites near exon.. Therefore, we only used meQTL pairs for the local random shift analysis.

3. **Visualization of recombination rate valleys**

Recombination rate values were converted to bedGraph format and then to bigwig format by UCSC tools “bedGraphToBigWig”. Custom perl script “alignWig2Bed.bigWigAverageOverBed.pl” with default options was used to extract average recombination rate from the bigwig files within each functional link. The average recombination values, e.g. Figure 1e-h, were smoothed and visualized by “permutation_random_plot.R” with default option. The scatter heatmap in Figure 2a was generated by “make_quantile_boxplot.pl” and “make_quantile_boxplot.R”. The global recombination rate in different window size was extracted by “alignWig2BedWindow.pl” with option “--window” to specify the window size. The individual genomic regions in Figure 1a-d were visualized by “plot_heatmap.local_norm.grey.pl” and “plot_heatmap.local_norm.grey.R” with different window sizes and bin sizes

4. **Processing of Hi-C signals**

Human Hi-C signal, normalized by KRnorm [2] from 1kb resolution dataset in GM12878 [3], was extracted by perl script “get_hic_freq.sparse.pl” in sparse line mode. Each pairs within the same 1kb window or adjacent 1kb are marked as “NA” value. Hi-C signal in each SNP-CpG pairs was assigned by the value within the segment that probes located at. For example, the Hi-C signal between SNP 1 (chr1, 12345) and CpG 2 (chr1, 45678) will be assigned to the Hi-C signal in pairs (chr1:12kb segment and chr1:45kb segment). The similar step was done for Hi-C in mouse CH-12 cells, but with 5kb window size. Only top 10% Observation/Expectation of the Hi-C links (O/E value is obtained directly from [3]) within 1Mb distance were kept for the analysis in main text. Hi-C links with CTCF motif in-between or nearby (<=+/-4kb) were excluded for the analysis. Bedtools v2.16.1 was used for the genomic intersection[4].

5. **Process of H2A.X, gamma H2A.X and anti-DMC1 ssDNA ChIP-seq data**

Raw reads of H2A.X and gamma H2A.X were mapped by bwa-0.7.10 [5] to hg19 genome with default options. Mapped reads of anti-DMC1 ssDNA ChIP-seq were obtained from the publication [6]**.** The Z score was calculated by “bam2normalizedwig.pl” as described in the previous work [1].

6. **Functional links overlapped with housekeeping genes and early developmental genes**

For each of functional link, if either end were within 1kb region of housekeeping gene annotation, it would be classified as overlapped with housekeeping genes. If neither end were within 1kb region of housekeeping gene annotation, it would be classified as not-overlapped with housekeeping genes. The same criterion was applied to measure the overlap with different early developmental genes. The details were implemented in perl script “separate_HK_gene_links.pl”.

7**. PRDM9 motif**

PRDM9 motif file was extracted from HOMER’s [7] default motif database. FIMO [8] in MEME 4.9.1 suite was used to calculate PRDM9 motif position with default options at reference genome within different functional links.

8**. Relationship between recombination rate, recombination hotspot density and DNA methylation**

Only functional links more than 10kb were used for the study. Region with recombination rate equals to 0 was marked as ‘NA’ in the analysis to avoid the artifact of no SNP representation in genetics map. Hotspot density was calculated by sum_of_hotspot_region_length/ functional_links_length. Only intervals with at least 10 CpGs (CpG locus in +/- strand are accounted as two separate CpGs) are used to estimate mean methylation level, otherwise, the value will be marked as ‘NA’ .

9**. Relationship between recombination rate, DNA methylation and DNA double stranded break initiation and repair efficiency**

Normalized H2A.X, gamma H2A.X and DSB initiation frequency (anti-DMC1 ssDNA ChIP-seq) was processed as Supplementary method 7 and generated bigwig files. These DSB values, recombination rate and DNA methylation in different window size were extracted by “alignWig2BedWindow.pl” with option “--window” to specify the window size. P value and correlation coefficient was calculated by cor.test() function in R-3.1.1.

10. **Correlation Based DNase-TSS Links**

10.1. Dataset selection

252 datasets (covering 110 cell types) with expression and DNaseI annotations were taken from [9]. A score was computed for each dataset as the correlation across all genes between expression and the presence of a DHS peak within 500bp of the TSS (DHS presence was z-score normalized, expression was quantile and z-score normalized; see below). Datasets whose score is less than 0.1 were discarded and at most one dataset was selected per cell type (breaking ties by score), resulting in 82 selected datasets.

10.2 Uniform DHS regions across cell types

A 150bp region was produced centered on each DHS region across all 82 selected datasets. Each region was scored by the number of cell types overlapping in the original input regions. Non-overlapping regions were selected in a greedy way based on this score (with ties ordered randomly).

10.3. Expression

For each of the 82 expression datasets, the average log expression across all probes for a given transcript was taken. Each dataset was quantile normalized against the others and the resulting expression values for each gene were z-score normalized.

10.4. Computing correlation

The Pearson correlation was computed between each gene’s normalized expression and all DHS regions within 1MB of the corresponding TSS. Control correlations that maintain chromosomal and distance biases were produced for various calculations. For each chromosome, all tested gene-DHS pairs were sorted by their distance. The DHS for each gene-DHS pair was then swapped between adjacent gene-DHS pairs in the sorted order. The resulting correlations from these pairs across all chromosomes was then used as control correlations, maintaining the distance and using it where appropriate.

10.5 Computing the linking modulation score (LMS)

For each gene and modulator (e.g. CTCF), the sites closest to either side of a TSS are used to partition the DHS into two sets: “inside” (proximal to the TSS) and “outside” (distal to the TSS). DHS directly overlapping the modulator site and genes with no modulator within 1MB are excluded from analysis. When comparing to other measures of linking (e.g. eQTLs or synteny conservation), the “outside” is defined as the DHS-TSS pairs that are included in the alternate measure, and other pairs are considered “inside”.

The correlations to inside vs. outside DHS regions cannot be directly compared because of biases in distance between the two sets. We correct for this confounder by sorting all correla- tions by distance (with ties broken randomly), and pairing the first inside correlation with the first subsequent outside correlation and repeating (alternating between first selecting an inside correlation and an outside correlation and discarding the interleaving correlations).

These pairs are then used to compute a mean difference in correlation. We define the “linking modulation score” (LMS) as this mean difference in correlation when linearly normalized with 0 being the difference between the observed and random correlations and 1 being no difference.

When performing multiple tests we adjust for spuriously high values by producing conservative estimates for the mean difference. The standard error under a normal assumption is used to produce a confidence interval around the mean difference (with α = 0.025/n where n is the number of tests) and the value within the confidence interval closest to no change is used for sorting the strength of each modulator.

11. **Correlation Based Enhancer-TSS Links**

We linked chromatin state defined enhancers with genes using data from the Roadmap Epigenomics project [10] and the method presented in [11] with a few small modifications. The method predicts links using chromatin state information, position of the enhancer relative to the TSS, and the correlation of multiple chromatin marks with gene expression across cell types. Here we used the correlation with gene expression of the signal of five chromatin marks: H3K27ac, H3K9ac, H3K4me1, H3K4me2, and DNaseI hypersensitivity. The gene expression data was the RPKM expression data for protein coding exons across 56 reference epigenomes from the Roadmap Epigenomics project (available in the file 57epigenomes.RPKM.pc from <http://compbio.mit.edu/roadmap>; Universal Human Reference was excluded). The chromatin mark signal was the -log10(p-value) tracks averaged to a 200-bp resolution. As input to our code we used the version of those tracks first averaged at 25-bp resolution using the ‘Convert’ command of ChromImputev [12]. In computing correlation between a specific chromatin mark signal and gene expression we used the Pearson correlation and omitted from the calculation samples lacking both chromatin mark signal and gene expression data. We made predictions separately for each of the 127 reference epigenomes and locations assigned to chromatin states, 6_EnhG, 7_Enh, and 12_EnhBiv, of the 15-state core 5-marks ChromHMM model[12, 13]. We restricted our predictions to chromatin state assignments on chr1-22 and chrX. We considered linking 200-bp bins within 1MB of a TSS of each gene as annotated in the file Ensembl_v65.Gencode_v10.ENSG.gene_info available from <http://compbio.mit.edu/roadmap>. If a gene had multiple TSS, then we only used the outermost TSS.

The method for linking is based on determining for each combination of cell type, chromatin state, and position relative to the TSS the estimated probability the set of correlations we observed would come from the actual data compared to randomized data. To this end we created a training set of actual observed correlations (positive examples) and correlations computed after randomizing which gene expression values were assigned to which genes (negative examples) separately for each combination of cell type, chromatin state, and position relative to the TSS. Each entry in the training set has five features corresponding to correlations for each of the considered chromatin marks. There is a positive and a corresponding negative entry for each instance of the specified chromatin state in the specified cell type at the specified position relative to the TSS or within 5kb of it (for smoothing purposes). We trained a logistic regression classifier to discriminate actual correlations with randomized correlations. We used the logistic regression library implemented in the Weka package version 3.7.3 with the regularization parameter set to 1 [14]. For considering linking a specific instance of a chromatin state assignment in a specific cell type and position relative to the TSS of a gene we applied the corresponding classifier. Let *p* denote the probability the classifier gives of being in the positive class of the actual observed correlations. We retained those links for which *p*/(1-*p*) was greater than or equal to 2.5. The method we used here is implemented in the code LinkingRM.java. Predictions are available at www.biolchem.ucla.edu/labs/ernst/roadmaplinking. For the analyses presented here we used those links for the primary enhancer state, 7_Enh.

12. **Recombination rate prediction within random genomic intervals**

10,000 random intervals were generated in the whole autosome regions. The length of these intervals was uniformly distributed within 10kb-100kb region. Random Forrest regression model was applied to predict the recombination rate at these random intervals by using chromosome number, genomic distance, the fraction of length overlapped with meQTL, the fraction of length overlapped with eQTL, the fraction of length overlapped with the top 10% of Hi-C links (O/E, no CTCF motif), the fraction of length overlapped with DNase-TSS links and average oocyte CpG methylation level within the intervals. The model was trained in 10,000 random intervals and then tested in another non-overlapped 10,000 random intervals. The Mean Square Error and predicted recombination value was calculated by randomForest function with option “importance=TRUE, na.action = na.omit, ntree=500” at “randomForest” library from CRAN in R-3.1.1. The same step was repeated 100 times to get the average and standard deviation of MSE, pearson correlation coefficient and spearman correlation coefficient. Also, the same steps were repeated for the random intervals in 100kb-1Mb region.

13. **Recombination rate prediction within functional links**

Random Forrest regression model was applied to predict the recombination rate within these functional intervals by using chromosome number, genomic distance, the hotspot density from 1000 Genome project and average oocyte CpG methylation level within the functional intervals. Ten fold cross validation was applied. The Mean Square Error and predicted recombination value was calculated by randomForest function with option “importance=TRUE, na.action = na.omit, ntree=500” at “randomForest” library from CRAN in R-3.1.1. The same steps were repeated for the random intervals in 100kb-1Mb region.

**Reference:**

1. Lay FD, Liu Y, Kelly TK, Witt H, Farnham PJ, Jones PA, Berman BP: **The role of DNA methylation in directing the functional organization of the cancer epigenome.** *Genome Res* 2015, **25:**467-477.

2. Knight PA, Ruiz D: **A fast algorithm for matrix balancing.** *Ima Journal of Numerical Analysis* 2013, **33:**1029-1047.

3. Rao SSP, Huntley MH, Durand NC, Stamenova EK, Bochkov ID, Robinson JT, Sanborn AL, Machol I, Omer AD, Lander ES, Aiden EL: **A 3D Map of the Human Genome at Kilobase Resolution Reveals Principles of Chromatin Looping.** *Cell* 2014, **159:**1665-1680.

4. Quinlan AR, Hall IM: **BEDTools: a flexible suite of utilities for comparing genomic features.** *Bioinformatics* 2010, **26:**841-842.

5. Li H, Durbin R: **Fast and accurate short read alignment with Burrows-Wheeler transform.** *Bioinformatics* 2009, **25:**1754-1760.

6. Pratto F, Brick K, Khil P, Smagulova F, Petukhova GV, Camerini-Otero RD: **DNA recombination. Recombination initiation maps of individual human genomes.** *Science* 2014, **346:**1256442.

7. Heinz S, Benner C, Spann N, Bertolino E, Lin YC, Laslo P, Cheng JX, Murre C, Singh H, Glass CK: **Simple combinations of lineage-determining transcription factors prime cis-regulatory elements required for macrophage and B cell identities.** *Mol Cell* 2010, **38:**576-589.

8. Grant CE, Bailey TL, Noble WS: **FIMO: scanning for occurrences of a given motif.** *Bioinformatics* 2011, **27:**1017-1018.

9. Thurman RE, Rynes E, Humbert R, Vierstra J, Maurano MT, Haugen E, Sheffield NC, Stergachis AB, Wang H, Vernot B, et al: **The accessible chromatin landscape of the human genome.** *Nature* 2012, **489:**75-82.

10. Roadmap Epigenomics C, Kundaje A, Meuleman W, Ernst J, Bilenky M, Yen A, Heravi-Moussavi A, Kheradpour P, Zhang Z, Wang J, et al: **Integrative analysis of 111 reference human epigenomes.** *Nature* 2015, **518:**317-330.

11. Ernst J, Kheradpour P, Mikkelsen TS, Shoresh N, Ward LD, Epstein CB, Zhang X, Wang L, Issner R, Coyne M, et al: **Mapping and analysis of chromatin state dynamics in nine human cell types.** *Nature* 2011, **473:**43-49.

12. Ernst J, Kellis M: **Large-scale imputation of epigenomic datasets for systematic annotation of diverse human tissues.** *Nat Biotechnol* 2015, **33:**364-376.

13. Ernst J, Kellis M: **ChromHMM: automating chromatin-state discovery and characterization.** *Nat Methods* 2012, **9:**215-216.

14. Witten IH, Frank E, Hall MA, Books24x7 Inc.: **Data mining practical machine learning tools and techniques, third edition.** In *Morgan Kaufmann series in data management systems*, 3rd edition. Burlington, Mass.: Morgan Kaufmann Publishers,; 2011.
